# Lipid hydroperoxides in nutrition, health, and diseases

**DOI:** 10.2183/pjab.97.010

**Published:** 2021-04-09

**Authors:** Teruo MIYAZAWA

**Affiliations:** *1 Food Biotechnology Platform Promoting Project, New Industry Creation Hatchery Center (NICHe), Tohoku University, Sendai, Miyagi, Japan.

**Keywords:** chemiluminescence, lipid hydroperoxides, rancid cooking oil, immunocompetent cells, atherosclerosis, Alzheimer’s disease

## Abstract

Research on lipid peroxidation in food degradation, oil and fat nutrition, and age-related diseases has gained significant international attention for the view of improvement of societal health and longevity. In order to promote basic studies on these topics, a chemiluminescence detection-high performance liquid chromatography instrument using a high-sensitivity single photon counter as a detector was developed. This instrument enabled us to selectively detect and quantify lipid hydroperoxides, a primary product of lipid peroxidation reactions, as hydroperoxide groups at the lipid class level. Furthermore, an analytical method using liquid chromatography–tandem mass spectrometry has been established to discriminate the position and stereoisomerization of hydroperoxide groups in lipid hydroperoxides. Using these two methods, the reaction mechanisms of lipid peroxidation in food and in the body have been confirmed.

## 1 Introduction

**Figure 1.**
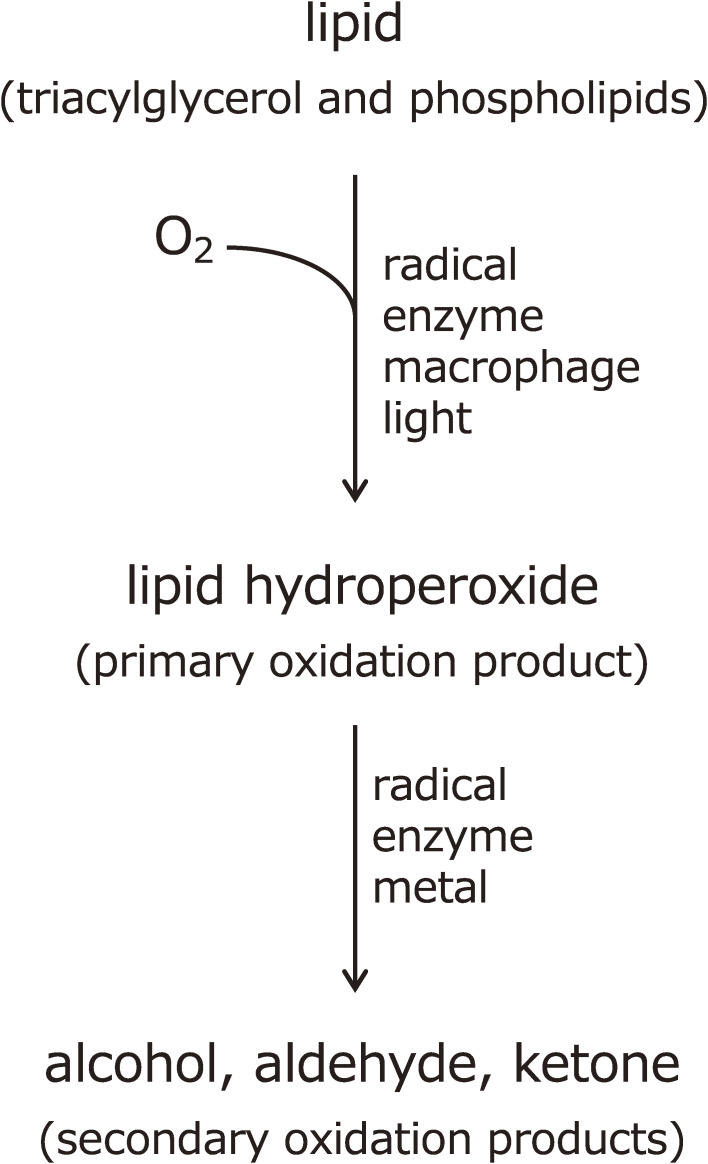
Lipid peroxidation reaction and its products.

The toxic nature of lipid hydroperoxides occurring in oxidized oil was first reported in the 1950s.^1–4)^ The biological and nutritional effects of lipid hydroperoxides have been investigated, but the analytical methodology for lipid hydroperoxide determination is very limited. When lipid molecules undergo a peroxidation reaction, a lipid hydroperoxide is formed as a primary oxidation product, and further secondary oxidation products, such as alcohols and aldehydes, are obtained as well (Fig. 1). As a doctoral student at Tohoku University circa 1980, the author began research and analysis of lipid peroxide formation in food and physiological effects in biological systems. At the time, the ingestion of rancid oil and/or membrane lipid peroxidation in tissue organs as the causes of lifestyle and age-related disease were beginning to attract international attention. However, in order to understand the mechanism of lipid molecule peroxidation, lipid hydroperoxides, which are the primary reaction product, had to be quantified selectively and with extreme sensitivity, which was not possible with conventional methods. Furthermore, the methodology was not well established internationally. At that time, analysis of volatiles by gas chromatography, ultraviolet (UV) absorption measurements, carbonyl values, peroxide value (PV), oxygen absorption methods, thiobarbituric acid methods, carotene bleaching, volatile low-molecular weight acids (mainly formic acid), and other methods were used. However, none of these analytical methods met our research objectives.

During a chemical reaction, a molecule absorbs the reaction energy and becomes an excited molecule. When transitioning to the ground state, most of the energy is released as thermal energy. A small fraction of molecules exhibit chemiluminescence (CL) associated with spontaneous photon emission from excited molecules. The theoretical sensitivity of this CL detection was estimated to be about 20,000 times higher than the fluorescence detection method, which was considered the most sensitive. We felt that this CL detection method is indeed suitable for the analysis of lipid hydroperoxides, which are ultra-traceable peroxide molecules that occur in the body. The properties of biological radical reactions by spontaneous photon counting were first examined from this background. Next, we developed a chemiluminescence-high performance liquid chromatography (CL-HPLC) system that applies this photon-counting device to the post-column position of HPLC, where lipid class levels of lipid hydroperoxides were successfully determined. We also established a method for the synthesis of lipid hydroperoxides as a standard required for accurate determination. The resulting high-purity lipid hydroperoxide standards have been provided to domestic and foreign researchers to support and develop lipid peroxide research. Recently, a lipid hydroperoxide quantification method that can discriminate the positional isomerism of hydroperoxide groups using state-of-the-art liquid chromatography–tandem mass spectrometry (LC-MS/MS) instrumentation has also been developed. These analytical methods and instruments have been used to advance quantitative research on the mechanisms of lipid peroxidation and its protection in food, nutrition, and disease, and this will form the content in this paper.

## 2 Single photon counting in evaluating nutritive value and toxicity of rancid cooking oil

By 1953, the principal toxic substance in oxidized oil had been identified as lipid hydroperoxide, which is a primary oxidation product in rancid edible oils.^5)^ Kaneda *et al.* observed that fish oil is rich in ω-3 polyunsaturated fatty acids, which become toxic once oxidized simply by being exposed to air. This study was groundbreaking in that it showed that while fish oil had been considered poisonous, in fact, fresh fish oil itself was not poisonous, unlike oxidized fish oil. It was also confirmed that rats fed with oxidized edible oil exhibited retarded growth with a decrease in body weight, as reviewed by Kaneda and Miyazawa.^6)^

**Figure 2.**
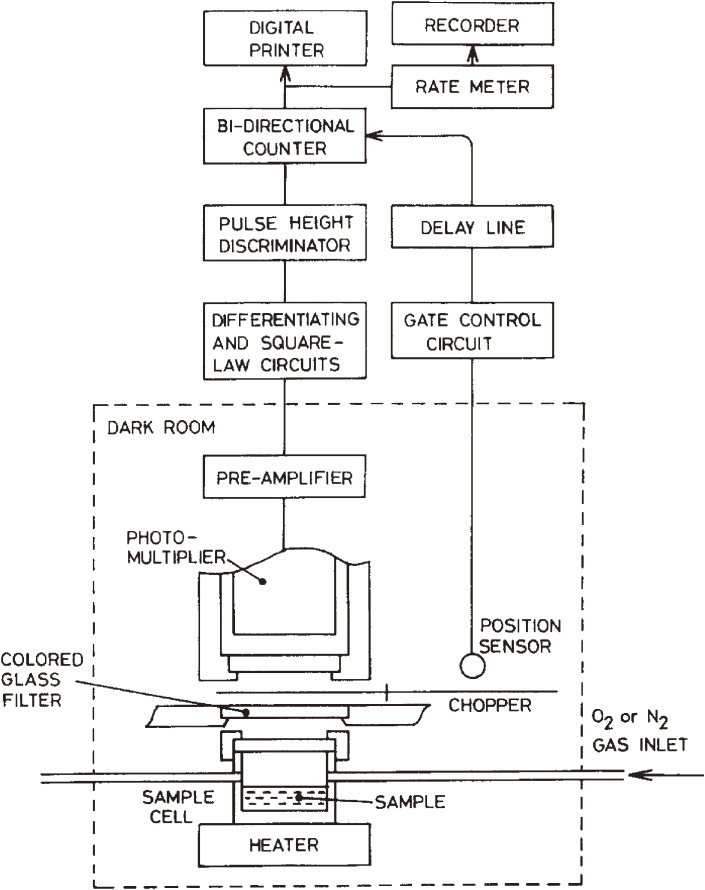
Schematic diagram of the ultra-high sensitivity photon counting system specially designed for biomedical and clinical applications. Adapted with permission from Ref. 7. Copyright 1982 Elsevier.

Nutritional experiments using such rats were of interest. First, to detect spontaneous low-level photon emission derived from electrically excited molecules that can occur in the liver of rats treated with rancid oil, a highly sensitive single photon counting system was developed (Fig. 2).^7)^ Using this device, low-level CL was successfully observed to elicit a radical reaction in the livers of rats orally administered with oxidized oil.^7–9)^ Stronger CL was also observed in the brain, liver, and blood of rats deficient in the antioxidant vitamin E, compared with normally fed rats.^8)^ We hypothesized that oxidized oil intake and antioxidant vitamin deficiency, inducing oxidative stress, increased radical production and induced lipid peroxidation in rat tissues.^9–11)^ In parallel with these studies, we have discovered that oral ingestion of oxidized oil causes severe damage to mouse immune tissues.^12)^ Namely, when graded amounts of oxidized oil were orally administered to C57BL/6 mice, necrosis was observed in lymphocytes located among the reticular network in the thymus, and thymus weight significantly decreased 24 h after the dosage of oxidized oil, together with decreases in spleen weight and blood leucocytes number.^13,14)^ These studies have prompted food manufacturers to devise improvements in food manufacturing processes that maximally limit the presence of lipid peroxides. It also showed that antioxidation of food fats and oils is an extremely important evaluation criterion for long shelf life and safe food development.

This spontaneous low-level CL analytical method was applied to the shelf life dating of fish in terms of oxidative rancidity and was used for the convenient assessment of lipid degradation in foods and food ingredients.^15)^ Edible fats and oils have strong CL intensity in the early stages of oxidation, and their emission spectrum showed that an excited oxygen molecule, singlet molecular oxygen (^1^O_2_), is produced by the decomposition of lipid hydroperoxide.^16)^ The formation of its reaction intermediates, oxygen-centered radicals such as lipid alkoxyl and peroxyl radicals, was confirmed using electron spin resonance (ESR) with a spin trap method.^17,18)^ If ^1^O_2_, a potent oxidant, occurs even at very low concentrations in the initial stage of fat oxidation, lipid peroxidation is induced, and lipid hydroperoxide accumulation leads to a pronounced oxidation-induced propagation stage. Vegetable and fish oils, which are high in polyunsaturated fatty acids, are more susceptible to such peroxidation. Because edible oils are relatively high in antioxidants, such as vitamin E and carotenoids, it was found that lipid peroxidation does not easily progress when an antioxidant is considered in the manufacturing process of edible oils.

*Drosophila melanogaster*, the fruit fly, has been used in many biological fields, such as aging research and mutagenicity testing. It has been established that the environmental temperature reflecting oxygen consumption can determine the life span of fruit fly.^19,20)^ Spontaneous photon emission has been measured as a monitor of free radical evolution in *D. melanogaster*, which had been maintained at 25 ℃ or 30 ℃ for 5 days after emergence.^21)^ When maintained at 30 ℃ the fly CL intensity was stronger than at 25 ℃. In higher temperature conditions, the fly life span was shorter (mean life span 29 days at 30 ℃ and 63 days at 25 ℃), and oxygen consumption (3.7 µL/mg.h at 25 ℃ and 4.9 µL/mg.h at 30 ℃) and mobility (movement distance was 25 mm/min at 25 ℃ and 700 mm/min at 30 ℃) increased, together with augmentation of lipid hydroperoxides in fly total lipids.^21)^ These findings suggested that as oxygen metabolism increases, the CL reactions that involve oxygen-dependent free radical metabolism, including membrane lipid peroxidation, contribute to the acceleration of fly body senescence. Spontaneous low-level CL was also confirmed in the bodies of flies fed with chemical mutagens, such as polycyclic aromatic hydrocarbon quinones and carcinogenic bracken fern.^22)^ Free radical formation was stimulated, as indicated by the enhanced CL in mutagen- or carcinogen-dosed flies, and, as a result, lipid hydroperoxide accumulation accompanied mutation, identified by the wing spot test in *D. melanogaster*.^21,22)^ The research employed CL monitoring and suggested that reactive oxygen species (ROS) and lipid radicals are involved in aging processes and mutagenicity.

## 3 Development of CL-HPLC for measuring lipid hydroperoxides in human plasma and tissue organs following aging and senescence

**Figure 3.**
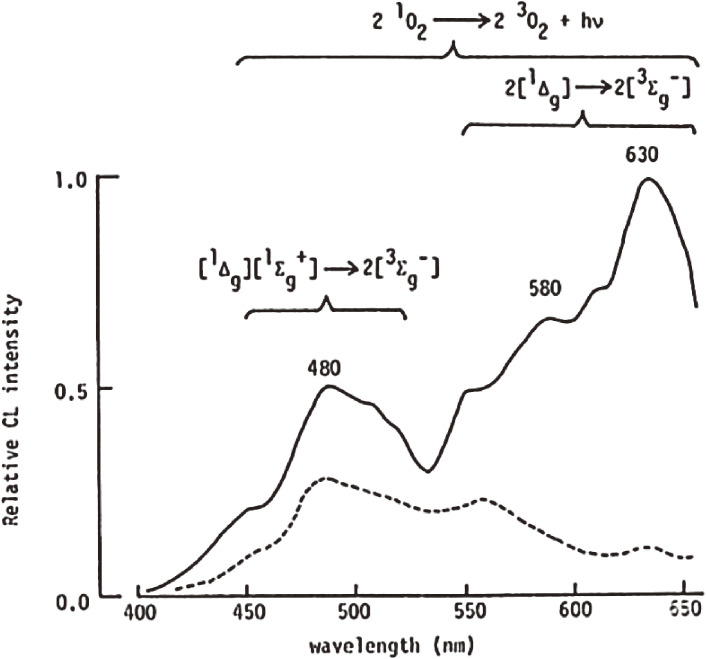
Chemiluminescence emission spectra observed between phosphatidylcholine hydroperoxide (PCOOH) and cytochrome *c*. The spectra were recorded using a filter spectral analyzer by mixing 1 M PCOOH with cytochrome *c* (1 µg/mL) in the presence (broken line) and absence (solid line) of 5 mM β-carotene (a singlet oxygen quencher). Adapted with permission from Ref. 26. Copyright 1990 John Wiley and Sons.

To trace the progression of lipid peroxidation in biological tissues, it is necessary to quantify lipid hydroperoxide, which is the primary product of lipid peroxidation reaction.^23,24)^ However, at the time, there was no analytical method that could sensitively and selectively quantify lipid hydroperoxides in living organisms. Therefore, we confirmed that lipid hydroperoxides can be detected by the CL method with good sensitivity.^25)^ The CL assay for the determination of lipid hydroperoxides is based on the detection of CL generated from the oxidation of luminol by the reaction with lipid hydroperoxides and cytochrome *c*.^25)^ Cytochrome *c* in the presence of lipid hydroperoxides is a favorable source for generating ROS, *i.e.*, ^1^O_2_ (Fig. 3), which oxidize luminol and emit lipid hydroperoxide-dependent CL. The CL reactions of lipid hydroperoxide with a reagent composed with cytochrome *c* and luminol are given in the following equations^26)^:

$$\begin{array}{rl} \underset{\text{(LOOH)}}{\text{lipid hydroperoxide}} & +\underset{\text{(heme)}}{\text{cytochrome }c}\\ & \to \text{lipid peroxyl radical} \end{array}$$ [1]

$$\text{2 lipid peroxyl radicals}\to {}^{1}\text{O}{}_{2}+\text{alcohol}+\text{ketone}$$ [2]

$${}^{1}\text{O}{}_{2}+\text{luminol}\to \text{luminol endoperoxide}$$ [3]

$$\begin{array}{rl} \text{luminol endoperoxide} & \to \text{excited aminophthalate}\\ & \;+\text{N}{}_{2}+hv\;\text{(430\textbackslash{},nm)} \end{array}$$ [4]

**Figure 4.**
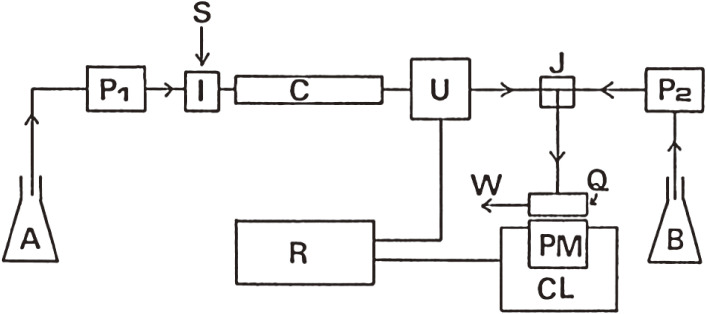
Schematic diagram of chemiluminescence detection-high performance liquid chromatography (CL-HPLC) for the lipid hydroperoxides assay. A, mobile phase; P, pump; I, sample injection valve; S, sample; C, column in a column oven; U, UV detector; J, mixing joint; B, chemiluminescence reagent consisting of cytochrome *c* and luminol in borate buffer; Q, spiral Teflon cell; PM, photomultiplier; CL, chemiluminescence detector; R, multiple recorder and integrator; W, waste. Adapted with permission from Ref. 26. Copyright 1990 John Wiley and Sons.

We developed a CL-HPLC system for the sensitive determination of lipid hydroperoxides, with specificity to hydroperoxide groups, using a CL reaction selective for lipid hydroperoxides as the detection part of HPLC (Fig. 4).^27)^ In this system, the CL detector was equipped with normal phase HPLC, and luminol-cytochrome *c* solution was used as the hydroperoxide-specific luminescent reagent. The detection limit was 7 nmol of phosphatidylcholine hydroperoxide (PCOOH). Using this CL-HPLC system, we revealed the presence of 28–431 pmol/ml of PCOOH in healthy human plasma.^26,28–30)^ Next, phospholipid hydroperoxides were quantified to verify the presence or absence of oxidative stress on membrane lipids in organs, such as the liver and brain of rats and mice. The quantification of phospholipid hydroperoxides in biological tissues is important to assess the degree of peroxidative damage to membrane lipids (mainly phospholipids). For this purpose, the optimal conditions for the simultaneous CL assay of PCOOH and phosphatidylethanolamine hydroperoxide (PEOOH) in rat liver and brain were determined. A CL-HPLC method that incorporates a mixture of cytochrome *c* and luminol as a post-column hydroperoxide-specific luminescent reagent was used.^31)^ An n-propylamine-bound silica column with a mixture of hexane-2-propanol-methanol-water 5 : 7 : 2 : 1 (by vol.) as an eluent was used to determine both PCOOH and PEOOH, which were separated from each other and from other lipids and lipid-soluble antioxidants. Using the established analytical conditions, it was confirmed that both PCOOH (average 1300 pmol/g liver and 110 pmol/g brain) and PEOOH (average 720 pmol/g liver and 350 pmol/g brain) are present in the liver and brain of 3-month-old SD rats.^32)^

Peroxidation of membrane phospholipids has been implicated as one of the basic mechanisms of age-related pathological changes.^33,34)^ Expiration of hydrocarbon gas by aged rats, conjugated diene absorption of tissue lipids upon aging, and age-related fluorescence pigment formation have been measured to follow oxidative events.^35,36)^ However, to accurately assess the degree of lipid peroxidation in biological membranes, the direct measurement of primary oxidation products, PCOOH and PEOOH, was most desirable. Then, age-related changes in PCOOH as an index for oxidative membrane lipid damage were determined by CL-HPLC. Brain and liver PCOOH content increased significantly with age in both male and female rats. The brain PCOOH content of male 18-month-old rats was 4.4 times that of 1-month-old rats, and that of female 18-month-old rats was 3.5 times that of 1-month-old females.^37)^ The liver PCOOH content of the male 18-month-old rats was 9.3 times that of the 1-month-old rats, and the content in female 18-month-old rats were 4.7 times as much as the 1-month-old rats.^37)^ The results indicated that oxidative deterioration, such as membrane phospholipid peroxidation, is prevalent in the membrane lipids of rat brain and liver due to aging.

**Figure 5.**
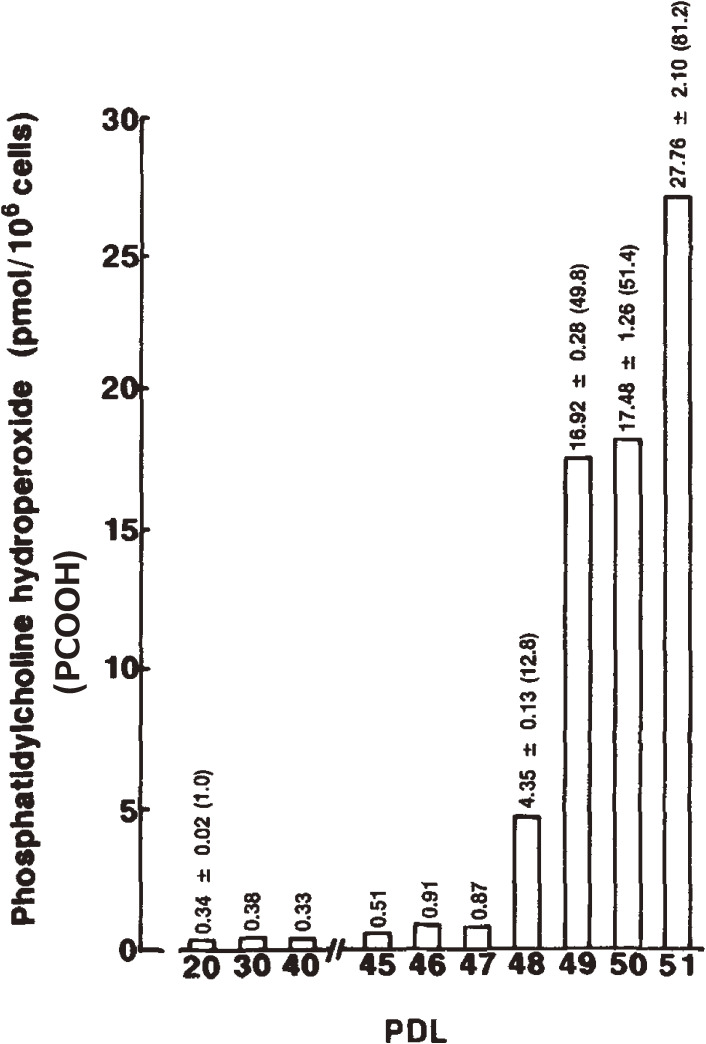
Changes in the phosphatidylcholine hydroperoxide (PCOOH) content per 10^6^ cells as a function of the population doubling level (PDL) of human fetal diploid. Values are the mean of three experiments, with SD. The figure in parentheses is the value relative to young cells (20th PDL). Adapted with permission from Ref. 38. Copyright 1993 John Wiley and Sons.

To understand age-dependent oxidative stress in cultured cells, the levels of PCOOH in serially cultured human fetal diploid fibroblasts (HE-1) at various population doubling levels (PDL) were determined by CL-HPLC.^38)^ The cellular PCOOH content increased with age from 0.34 to 27.72 pmol/10^6^ cells. At the end of the cells’ *in vitro* lifespan (51st PDL), the hydroperoxide content per 10^6^ cells reached about 80 times the level found in cells of the 20th PDL (Fig. 5). Supplementation with exogenous α-tocopherol as an antioxidant to the culture medium prevented PCOOH accumulation, but it did not extend lifespan *in vitro*. About 1.0–8.6% of α-tocopherol was incorporated into the cells, depending on the concentrations added to the medium.^38)^ Accumulation of cellular PCOOH with serial subculturing was related to a decrease in the cellular proliferation rate. The results indicated that substantial intracellular phospholipid hydroperoxide accumulation occurred throughout the course of human diploid fibroblast aging. According to the original concept of Hayflick and Moorhead,^39,40)^ the limited *in vitro* lifespan of cells was characteristic of phase III, which is the period of apparent decrease in the rate of cell proliferation accompanied by morphological, biochemical, and cytological changes. Our results revealed that oxidative deterioration of membrane lipids associated with membrane phospholipid peroxidation contributes to cellular aging.^38–46)^

PCOOH and PEOOH concentrations were determined in microsomes and plasma membranes prepared from 2- and 17-month-old male SD rat hepatocytes, to verify the dissimilarity in age dependency of lipid peroxidation in organelle membranes.^47)^ The hydroperoxides were measured by CL-HPLC, and 1-palmitoyl-2-(13-hydroperoxy-*cis*-9, *trans*-11-octadecadienoyl)phosphatidylcholine (PLPC-OOH) and 1-palmitoyl-2-(13-hydroperoxy-*cis*-9, *trans*-11-octadecadienoyl)phosphatidylethanolamine (PLPE-OOH) were enzymatically synthesized and utilized as standards for calibration. Baseline concentrations of hydroperoxides (PCOOH + PEOOH) of the 17-month-old rats were 46 pmol per mg protein in microsomes (2.7 times higher than in 2-month-old rats) and 306 pmol per mg protein in plasma membranes (9.9 times higher than in 2-month-old rats) (Table 1). In *in vitro* systems, both microsomal and plasma membrane lipids were severely oxidized and converted to PCOOH and PEOOH by NADPH-dependent lipid peroxidation, but age-dependency was only observed in the plasma membranes. These results demonstrated the substantial oxidative damage to membrane lipids that occurs with aging in microsomes and plasma membranes, but were more prevalent in plasma membranes richer in polyunsaturated phospholipids than in microsomes in rat hepatocytes.^47)^ It was also confirmed that the ingestion of excess amounts of docosahexaenoic acid (DHA, 22:6n-3) oil enhances lipid peroxidation in target membranes, such as in liver microsomes, where greater amounts of n-3 fatty acids are incorporated into microsomal phospholipids, when rats were fed 15% (w/w) DHA oils for up to 3 weeks.^48)^ This result revealed that excessive intake of highly unsaturated fatty acids and the unsaturation of membrane phospholipids increases the risk of oxidative stress disorders.

## 4 CL-HPLC of triacylglycerol (TG) hydroperoxides (TGOOH), hydrogen peroxide, and lipid ozonation products

**Figure 6.**
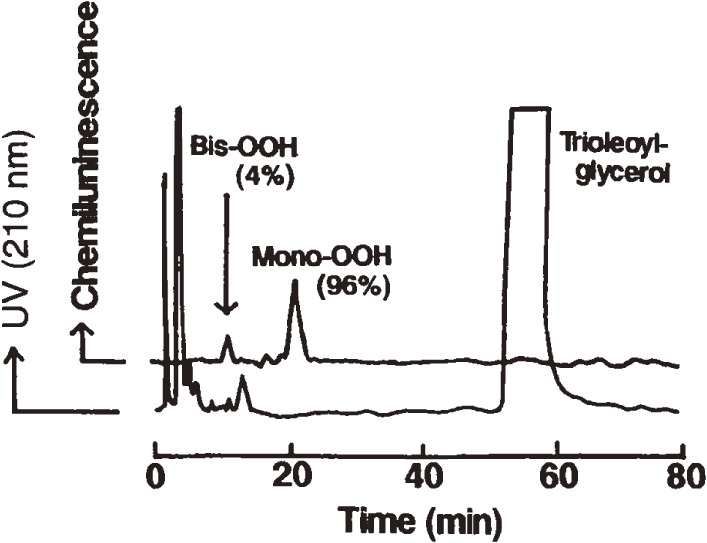
Chemiluminescence detection-high performance liquid chromatography (CL-HPLC) of trioleoylglycerol (peroxide value 0.16 meq/kg). The HPLC column was a Finepak SIL C18-5 (5 µm, 250 × 4.6 mm) connected with a Finepak SIL C18 T-P pre-column (5 µm, 50 × 4.6 mm). The mobile phase was methanol, and the flow rate was 1.1 mL/min. The chemiluminescence reagent was composed of cytochrome *c* and luminol. Mono-OOH, mono-hydroperoxides (retention time 21.0 min); Bis-OOH, bis-hydroperoxides (retention time 10.0 min); UV, ultraviolet. Adapted with permission from Ref. 49. Copyright 1995 John Wiley and Sons.

**Figure 7.**
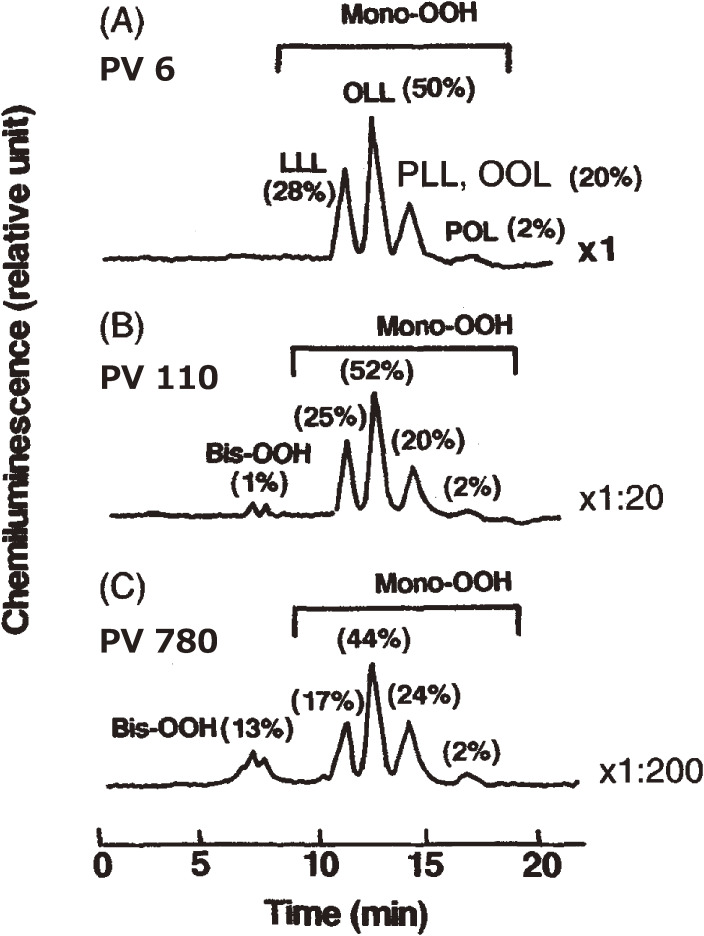
Chemiluminescence detection-high performance liquid chromatography (CL-HPLC) chromatograms of soybean oil (A, peroxide value [PV] 6 meq/kg; B, PV 110 meq/kg; C, PV 780 meq/kg) autoxidized at 25 ℃. Indicators, ×1, ×1 : 20, ×1 : 200, are the relative dilution ratios of oxidized oil. LLL, OLL, PLL, OOL, and POL depicted the fatty acid combination in the molecular species of triacylglycerol, with abbreviations representing palmitic (P), oleic (O), and linoleic (L) acids. Adapted with permission from Ref. 49. Copyright 1995 John Wiley and Sons.

Oxidation of food lipids and cooking oils causes undesirable tastes and rancid flavors. Ingestion of rancid oils has deleterious effects, especially on immunocompetent cells in mammals. Therefore, it is important to detect peroxides present in oils. A reliable method was needed to analyze the molecular species of oxidized vegetable oils. To accomplish this goal, mono-, bis-, and tris-hydroperoxides (Mono-OOH, Bis-OOH, and Tris-OOH, respectively) of TGs formed during autoxidation and photosensitized oxidation of oils were determined by CL-HPLC equipped with a reversed-phase column.^49)^ Mono-OOH was the major species (96% of total hydroperoxides) in trioleoylglycerol (18 : 1–18 : 1–18 : 1) [peroxide value (PV) 0.16 meq/kg] (Fig. 6), and Bis-OOH and Tris-OOH showed prolonged accumulation upon photo-oxidation. This profile was also confirmed by photo-oxidation of trilinoleoylglycerol (18 : 2–18 : 2–18 : 2) and trilinolenoylglycerol (18 : 3–18 : 3–18 : 3). Soybean oil (PV 6 meq/kg) contained Mono-OOH of oleoyl-linoleoyl-linoleoylglycerol (18 : 1–18 : 2–18 : 2) as the main peroxidic molecular species (50% of total hydroperoxides) (Fig. 7). Mono-OOH of trilinoleoylglycerol was the principal species (61% of total hydroperoxides) in safflower oil (PV 5 meq/kg), and Mono-OOH of oleoyl-oleoyl-linoleoylglycerol (18 : 1–18 : 1–18 : 2) was the representative species (66% of total hydroperoxides) in olive oil (PV 3 meq/kg).^49)^ These results showed that the molecular species of Mono-OOH formed during oxidation of vegetable oils depended on their TG composition. Bis-OOH represented 1% of the total hydroperoxides in olive oil, and Tris-OOH was not detected in either of the sample oils. The high sensitivity of the CL-HPLC method is beneficial to study the peroxidation mechanism and flavor changes in vegetable oils. On the other hand, CL-HPLC with gel permeation chromatography column effectively gives a single TGOOH peak without fatty acid composition influence.^50)^ TGOOH was calculated to be as low as PV 0.04 meq/kg, and this method is useful for studying mechanisms, especially of initial rancid reactions in edible oils.

Hydrogen peroxide (H_2_O_2_) is an oxidizing agent that has been used in sterilization and bleaching of food products, and H_2_O_2_ is speculated to occur in food processing. A CL-HPLC method was established for the determination of H_2_O_2_ at picomolar levels using a cation-exchange gel column.^51)^ At the time, HPLC analysis of H_2_O_2_ was considered difficult, because H_2_O_2_ was adsorbed by the column packing resin. We found out that a gel column with distilled water as the mobile phase allowed good separation of H_2_O_2_ without causing any irreversible binding of H_2_O_2_ to the column resin. The detection limit and the quantification limit of H_2_O_2_ were 4 and 6–600 pmol, respectively. The suitability of the present method was verified by the determination of H_2_O_2_ present in coffee drinks, and H_2_O_2_ content was estimated to be 67–165 µM (Table 2).^51)^

**Figure 8.**
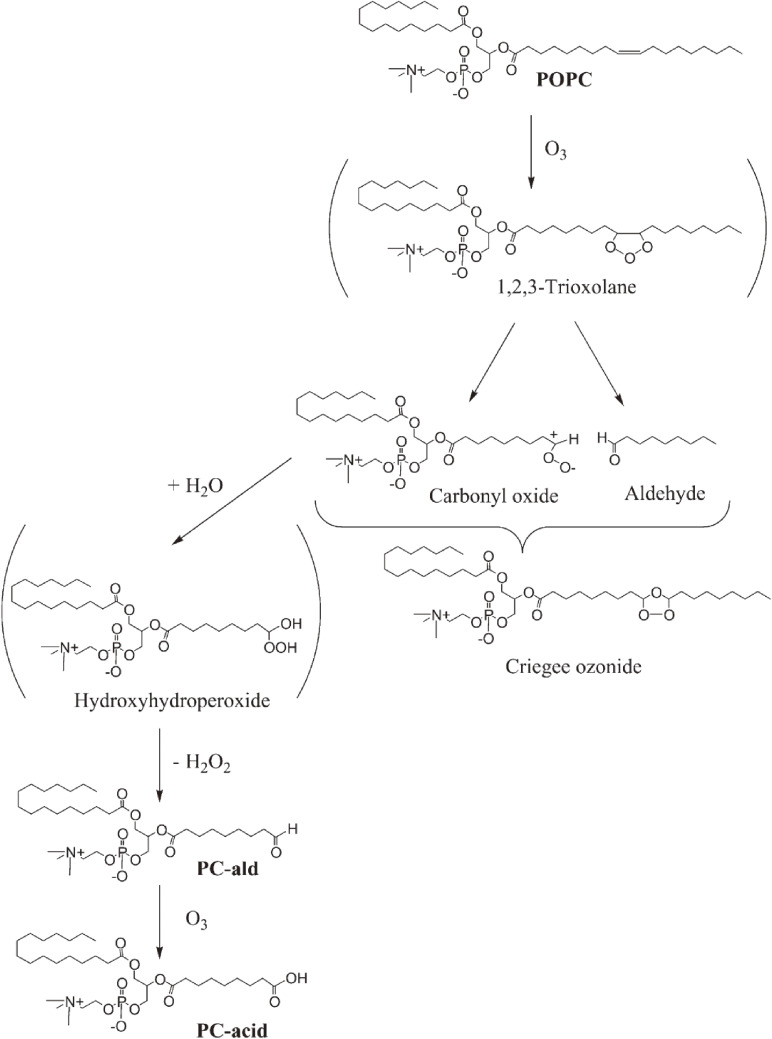
Chemical reaction of 1-palmitoyl-2-oleoyl-*sn*-glycero-3-phosphocholine (POPC) with ozone. PC-ald, 1-palmitoyl-2-(9-oxononanoyl)-*sn*-glycero-3-phosphocholine; PC-acid, 1-palmitoyl-2-(9-carboxynanoyl)-*sn*-glycero-3-phosphocholine. Adapted with permission from Ref. 56. Copyright 2002 John Wiley and Sons.

**Figure 9.**
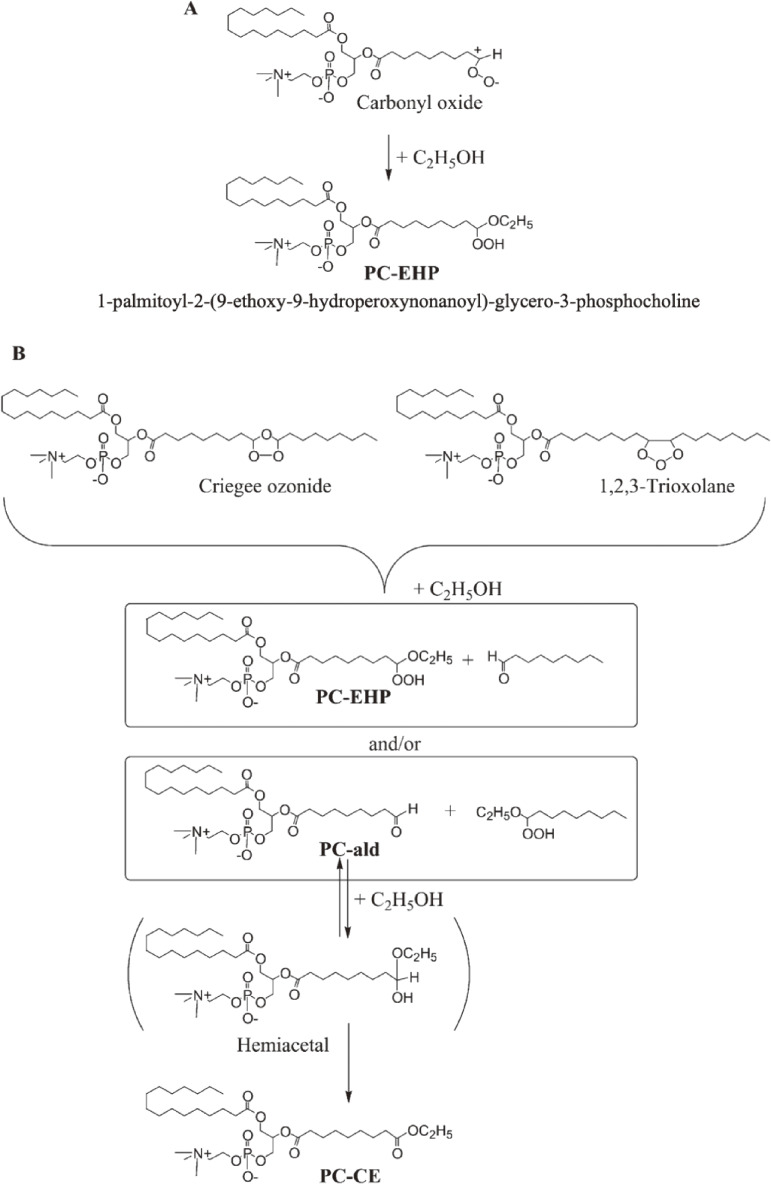
Proposed pathway for the formation of (A) phosphatidylcholine ethoxyhydroperoxide (PC-EHP) and (B) PC-CE during the ozonation of 1-palmitoyl-2-oleoyl-*sn*-glycero-3-phosphocholine (POPC) in ethanol. For abbreviations, see Fig. 8. Adapted with permission from Ref. 56. Copyright 2002 John Wiley and Sons.

**Figure 10.**
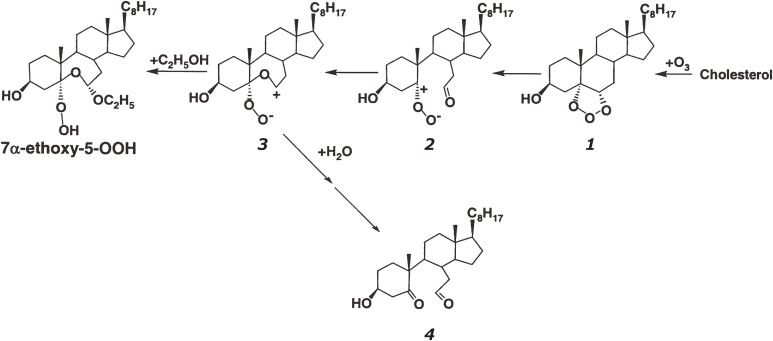
Formation of 7α-ethoxy-5-OOH (7α-ethoxy-3β-hydroxy-5α-B-homo-6-oxacholestene-5-hydroperoxide) during cholesterol ozonation in the presence of ethanol. Adapted with permission from Ref. 57. Copyright 2004 John Wiley and Sons.

Ozone, a powerful oxidant, is frequently used for disinfection, deodorization, and bleaching of wastewater and polluted air as well as foodstuffs, especially for the sterilization of marine products.^52)^ Excess exposure of animals and humans to ozone causes lung injury, including increased epithelial macromolecular permeability and neutrophil infiltration.^53)^ Because unsaturated lipids, *i.e.*, phospholipids and cholesterol, are recognized as ozone targets in cell membranes and foods, the reaction between ozone and lipids is of interest.^54,55)^ For the sterilization of foods, a combination of different disinfection procedures, *i.e.*, treatment with ozone together with alcohol or UV light, was used to enhance bactericidal effects. However, although oxidative damages are suspected in cellular lipids, the ozonation products of membrane phospholipids have never been confirmed as direct evidence. Then, the ozonation of 1-palmitoyl-2-oleoyl-*sn*-glycero-phosphocholine (POPC) in ethanol-containing solvent was analyzed by CL-HPLC with on-line electrospray mass spectrometry (MS) and characterized based on NMR spectroscopy and MS in high-resolution fast atom bombardment mode.^56)^ The reaction yielded a large amount of a novel ethoxyhydroperoxide compound [1-palmitoyl-2-(9-ethoxy-9-hydroperoxynonanoyl)-*sn*-glycerocholine], which is a potentially reactive ozonized lipid found in food and biological tissues.^56)^ The chemical reaction of POPC with ozone (Fig. 8) is summarized and proposed as a pathway for the formation of phosphatidylcholine ethoxyhydroperoxide (PC-EHP) and further ozonation products (Fig. 9). A unique ethoxyhydroperoxide molecule (7α-ethoxy-5-OOH, 7α-ethoxy-3β-hydroxy-5α-B-homo-6-oxacholestane-5-hydroperoxide) was confirmed as the main ozonation product of cholesterol in the presence of ethanol.^57)^ As shown in Fig. 10, the ozonation of cholesterol gave a primary ozonide 1, and the ozonide was converted into carbonyl oxide intermediate 2. The intramolecular partial capture of 2 by the 6-carbonyl oxygen yields the dipolar intermediate 3. Ethanol can readily react with intermediate 3 to form 7α-ethoxy-5-OOH. In the presence of water, intermediate 3 reacted with H_2_O, which gives secoaldehyde 4 (Fig. 10). This ethoxyhydroperoxide (7α-ethoxy-5-OOH) showed remarkable cytotoxicity toward human adenocarcinoma A549 cells, and its cytotoxicity was superior to that of the autoxidized products of cholesterol.^57)^

## 5 Squalene (SQ) hydroperoxides found in sunlight-exposed human skin

**Figure 11.**
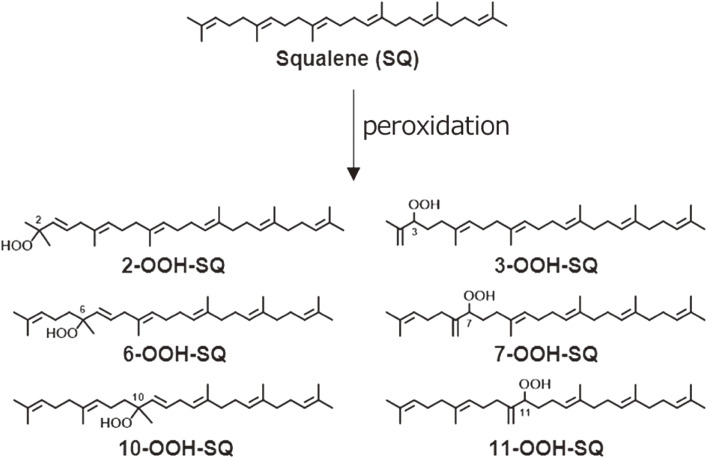
Chemical structures of squalene (SQ) and six monohydroperoxide (SQOOH) isomers formed by sunlight exposure. Adapted from Ref. 62.

Human skin is the largest organ of the body and is exposed constantly to sunlight stress, including UV light irradiation. The skin is rich in lipids consisting of SQ, TG, wax esters, and sterols; therefore, skin lipids are vulnerable to oxidative stress from sunlight. SQ is a structurally unique triterpene compound with six double bonds, and it seems to be the principal target for peroxidation at the skin surface. SQ hydroperoxide (SQOOH), as a primary oxidation product of SQ, was first detected by CL-HPLC with a reversed-phase column, and then the detection limit was as low as 1 pmol.^58)^ Skin surface lipids from sebum, the scalp, and dandruff were submitted for analysis. After exposure to sunlight or washing with shampoo, SQOOH could be detected in all samples examined. This confirmed that SQ is the first target lipid in the human skin surface that incurs oxidative stress by sunlight exposure.^58,59)^ Pure SQOOH isomers were prepared, and an analytical method for SQOOH isomers using a quadrupole/linear ion-trap mass spectrometer (QTRAP) MS/MS system was developed.^59,60)^ Collision-induced dissociation produced specific fragment ions for each SQOOH isomer, which permitted discrimination between SQOOH isomers by multiple reaction monitoring (MRM). When a lipid extract from human forehead skin was subjected to LC-MS/MS with MRM, individual SQOOH isomers could be separated and detected with a sensitivity of 0.05 ng/injection (Fig. 11). The total concentration of SQOOH isomers in forehead skin was about 950 µg/g skin lipids, but it increased up to 2,760 µg/g skin lipids after 3 h of sunlight exposure.^59,60)^ We further confirmed that SQOOH induces inflammatory responses in immortalized human keratinocytes.^61)^ SQOOH caused an increase in the expression of inflammatory genes, such as the interleukins and cyclooxygenase-2 (COX-2). Consistent with the upregulation of COX-2 mRNA, SQOOH enhanced ROS generation, nuclear factor kappa B activation, COX-2 protein expression, and prostaglandin E2 production.^61,62)^ On the other hand, tocotrienol (the unsaturated form of vitamin E) ameliorated SQOOH actions.^61)^ The findings indicated that SQOOH plays an important role in inflammatory skin disorders after exposure to excess sunlight.

**Figure 12.**
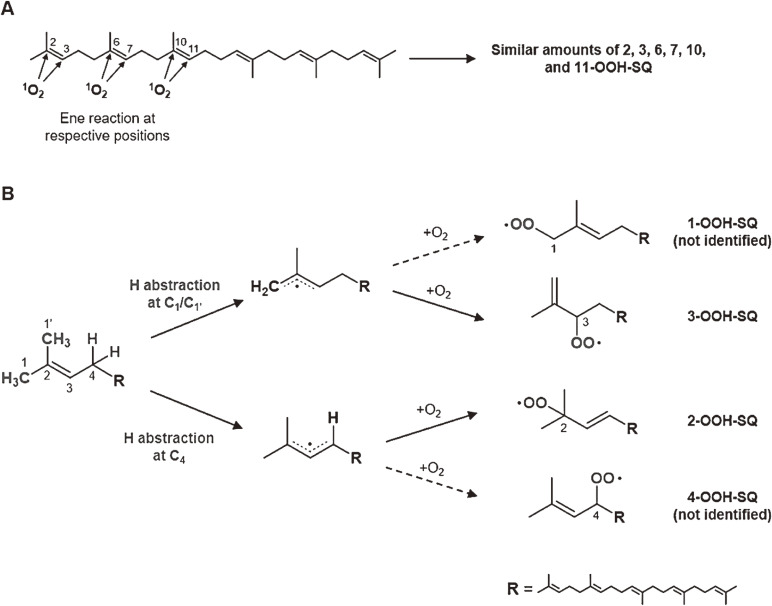
Predicted pathways of the singlet oxygen oxidation of squalene (SQ) (A) and free radical oxidation of SQ (B). Adapted from Ref. 62.

Oxidation of SQ causes a decline in the nutritional value of SQ in foods, as well as an accumulation of SQ oxidation products in skin lipids, which leads to adverse skin conditions. However, mechanistic insights into SQ oxidation by different mechanisms have been limited, and thus effective measures towards the prevention of SQ oxidation have not been identified. We oxidized SQ using either ^1^O_2_ oxidation or free radical oxidation and monitored the formation of the six SQ monohydroperoxide (SQOOH) isomers, the primary oxidation products of SQ, at the isomeric level (Fig. 11). Although ^1^O_2_ oxidation of SQ resulted in the formation of similar amounts of the six SQOOH isomers, free radical oxidation of SQ mainly formed two types of isomers, 2-OOH-SQ and 3-OOH-SQ (Fig. 12). The addition of β-carotene during ^1^O_2_ oxidation and the addition of α-tocopherol during free radical oxidation led to a dose-dependent decreases in the formation of SQOOH isomers.^62)^ Such results suggested that analysis of SQOOH at the isomeric level can allow the determination of the cause of SQ oxidation in various samples and provides a foothold for future planning of SQ oxidation prevention.

## 6 Plasma PCOOH in hyperlipidemia, atherosclerosis, diabetes, and antioxidant functions of green tea catechins

Hyperlipidemia is a major risk factor for the development of atherosclerosis.^63–65)^ A recognized pathophysiological factor, apart from cholesterol, is the formation of foam cells by the conversion of monocytes/macrophages through the scavenger pathway.^66)^ The process of foam cell formation is speculated to involve the oxidative modification of low-density lipoprotein (LDL).^67)^

**Figure 13.**
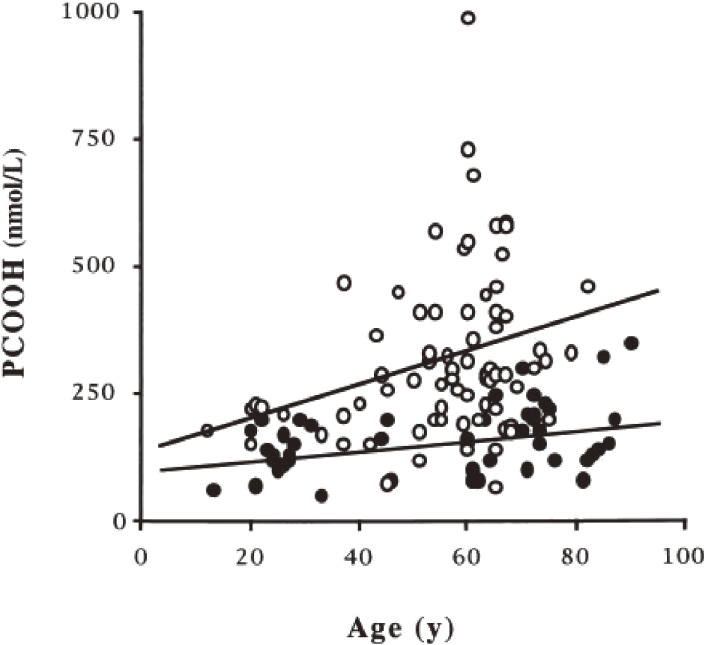
Correlation between plasma phosphatidylcholine hydroperoxide (PCOOH) and age. ●, control subjects (*r* = 0.392; *P* < 0.01); ○, patient with hyperlipidemia (*r* = 0.298; *P* < 0.01). Adapted with permission from Ref. 70. Copyright 2000 Oxford University Press.

**Figure 14.**
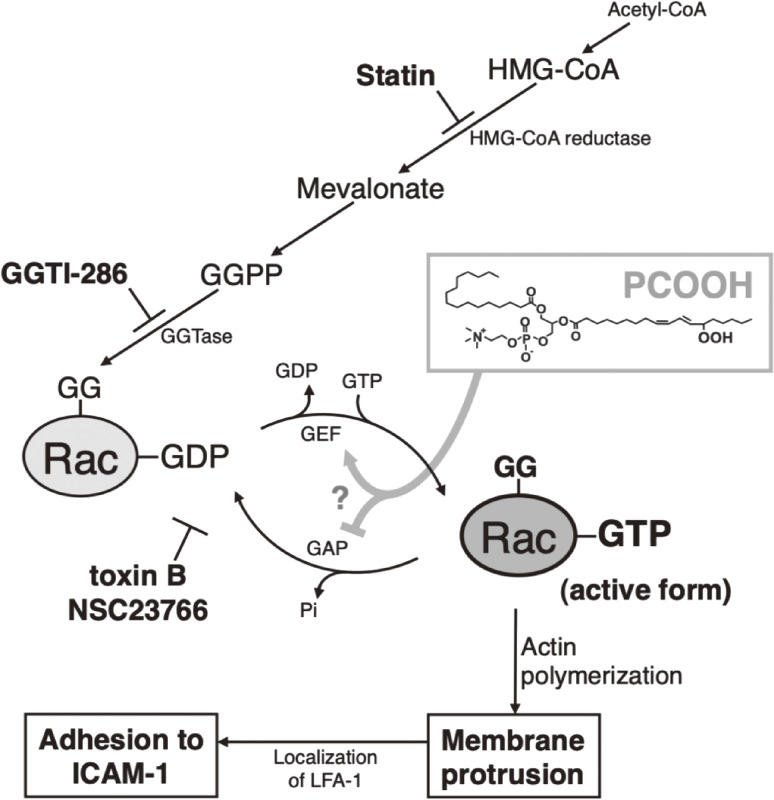
Involvement of Rac activation in phosphatidylcholine hydroperoxide (PCOOH)-induced THP-1 monocytic cell adhesion to intracellular adhesion molecule-1 (ICAM-1) in atherogenicity.

Phosphatidylcholine (PC) is a principal phospholipid on the surface of lipoprotein particles; therefore, we first tried to explain the occurrence of PCOOH as the ideal target molecule for clinical investigation.^68,69)^ Hyperlipidemic patients (44 males and 50 females, average age 56 years) and normolipidemic volunteers (controls, 32 males and 15 females, average age 55 years) were recruited, and their plasma PCOOH was determined using CL-HPLC.^70)^ Plasma PCOOH concentrations increased with age in both the controls and hyperlipidemic patients (Fig. 13). The mean plasma PCOOH concentrations in hyperlipidemia (331 nmol/L; n = 94) were significantly higher than in the control group (160 nmol/L; n = 47) (Table 3). Plasma PCOOH concentrations were similar in three hyperlipidemic phenotypes, hypercholesterolemia (IIa) and (IV), and combined hyperlipidemia (IIb). There was no correlation between plasma PCOOH and total cholesterol, triglycerides, or phospholipids in hyperlipidemic patients. For all subjects, there was a significant positive correlation between plasma PCOOH and each lipid class (total cholesterol, triglycerides, and phospholipids). The findings indicated that oxidative stress causing PCOOH formation in plasma lipoproteins in hyperlipidemia is a factor of the progression of atherosclerosis.^70)^ Oxidized LDL, rich in PCOOH as circulating oxidants, causes pancreatic β-cell dysfunction for insulin secretion and reduced preproinsulin mRNA expression in the β-cell-derived cell line HIT-T15.^71)^ We also confirmed that the plasma concentration of PCOOH increased in cholesterol-fed rabbits and found that fructose (fruit sugar that causes the Maillard reaction more easily than glucose) ingestion promotes this process and aggravates atherosclerosis.^72)^ To further estimate the atherogenicity of PCOOH, we evaluated the effect of PCOOH on THP-1 monocyte cell adherence to immobilized vascular endothelial cell adhesion molecules. It was confirmed that THP-1 cell adhesion to intracellular adhesion molecule-1 (ICAM-1) was dose-dependently increased by PCOOH. In PCOOH-treated cells, obvious protruding F-actin-rich membrane structures were formed, and lymphocyte function-associated antigen-1 (LFA-1) was localized to the protruding structures. Cytochalasin D, an actin polymerization inhibitor, suppressed PCOOH-induced cell adhesion to ICAM-1 and the membrane protrusions. These findings indicated that PCOOH evokes LFA-1-mediated cell adhesion to ICAM-1 via actin cytoskeleton organization, and this mechanism is recognized to participate in monocyte adherence to the arterial wall in the initiation of atherosclerosis.^73)^ Furthermore, we investigated the involvement of Rho family GTPases in PCOOH-induced THP-1 cell adhesion to ICAM-1.^74)^ Isoprenoid depletion by fluvastatin and geranylgeranyltransferase inhibition by GGTI-286 suppressed PCOOH-induced cell adhesion to ICAM-1 and F-actin-rich membrane protrusion formation. Pull-down assays demonstrated the activation of Rac1 and Rac2 in PCOOH-treated cells. Pan-Rho family GTPase inhibitor *Clostridium difficile* toxin B, Rac-specific inhibitor NSC23766, and RNA interference of the Rac isoforms suppressed cell adhesion. These findings indicated the involvement of Rac GTPase activation in PCOOH-induced cell adhesion to ICAM-1 via actin polymerization and reorganization (Fig. 14).^74)^ The modulation of Rho family GTPase activities provides novel insights into the pathophysiological consequences of phospholipid oxidation, because the Rho family GTPase-dependent action cytoskeleton organization plays a crucial role in various cellular processes, including adhesion, migration, phagocytosis, and exocytosis.^75–77)^

The role of PCOOH in the induction of angiogenesis was further investigated. Angiogenesis, the formation of new blood vessels, is observed in advanced atherosclerotic lesions. We examined the effect of PCOOH and found that PCOOH stimulates angiogenic responses (*e.g.*, vascular endothelial growth factor-induced cell proliferation, migration, and tube formation, and angiogenesis-related gene/protein expression) in human umbilical vein endothelial cells and an *ex vivo* rat aorta model (aortic ring assay).^78)^ The angiogenic effects of PCOOH are mediated via generation of ROS and activation of both the PI3K/AKT (PDK and AKT) and the mitogen-activated protein kinase (ERK, JNK, and p38) pathways. The findings indicated at first that PCOOH can elicit several angiogenic responses and is an important factor in atherosclerosis progression and plaque instability.

In patients with type 2 diabetes (DM), we first confirmed the increase in serum PCOOH concentration dependent on glycemic control.^79)^ Patients with DM (n = 61) and normal controls (n = 11) were enrolled, and high-density lipoprotein (HDL) and non-HDL (containing very-low-density lipoprotein, LDL, and a small amount of intermediate-density lipoprotein) were separated from serum, and PCOOH levels were determined using CL-HPLC. We found that (1) serum and non-HDL PCOOH increases in patients with DM, (2) the levels were strongly correlated with diabetes control, and (3) approximately half the amount of serum PCOOH was present in HDL in both controls and patients with DM. The study showed the significant increase in non-HDL PCOOH in patients with DM, which is important in DM atherogenicity. Approximately 50% of serum PCOOH is distributed in the HDL of both patients with DM and controls; however, as a general consideration, HDL might be more resistant to oxidative stress than LDL. HDL is recognized as the main carrier of lipid hydroperoxides in lipoproteins, which can be detoxified in the liver. The distribution ratio of PCOOH in HDL was not so different between the controls and patients with DM. Along with an increase in glycated hemoglobin (HbA_1c_) level, non-HDL PCOOH also increased. The pattern of PCOOH distribution in non-HDL and HDL fractions was approximately equal between lower and higher HbA_1c_ groups of patients with DM. The increase in serum PCOOH reflected the increase in the non-HDL fraction rather than the HDL fraction. Transfer of PCOOH from LDL to HDL or from peripheral tissue to HDL has been recognized. When PCOOH was studied in familial hyperalphalipoproteinemia (heterozygous deficiency of cholesterol ester transfer protein), serum PCOOH levels increased markedly due to increased HDL PCOOH. The data indicated that we have to improve hyperglycemia and normalize the HbA_1c_ level of patients with DM to decrease oxidative stress, as shown by PCOOH.^79–83)^

The elimination of plasma PCOOH was evaluated as a possible approach to lower the incidence of atherosclerosis. At first, we tried to evaluate the antioxidant function of a seleno-organic compound, ebselen [2-phenyl-1,2-benzoisoselenazol-3(*2H*)-one], which was expected to show glutathione peroxidase-like activity *in vitro*.^84)^ In an oral administration of ebselen in rats, plasma PCOOH showed a significant decrease at 2 h after the administration.^85)^ Such *in vivo* effects on plasma PCOOH elimination depended on both the dose and time after administration. The maximum decrease in PCOOH appeared around 2–4 h after oral administration, indicating that ebselen is a potent antioxidant for lowering plasma PCOOH.

Although ebselen was a candidate compound for a drug, we thought that food intake of antioxidants, such as those that reduce the increase in PCOOH in the blood, might be more desirable from the perspective of disease prevention, such as atherosclerosis. As the Japanese nation increases its longevity, there has been interest from abroad in the health functions of the Japanese diet. Therefore, the biological antioxidant effect of catechins contained in green tea, which is consumed with most meals, were evaluated. At that time, there were many studies focused on green tea catechins, but there were no analytical methods to detect catechins with high sensitivity and specificity, so we applied the CL method. An emission spectrometric analysis of catechins CL and polyphenols was carried out in the presence of hydrogen peroxide, acetaldehyde, and horseradish peroxidase as the CL reagent.^86)^ The analysis confirmed that the maximum emission wavelengths (Emax) strictly differed among catechins (Emax 630 nm), theaflavins (Emax 690 nm), and anthocyanins (Emax 675 nm), reflecting their chemical structures, as follows (Table 4):

$$\begin{array}{rl} & \text{P (polyphenol)}+\text{HOOH}\\ & \;\to \text{PH-OOH (oxygenated intermediate }\\ & \;\text{of polyphenol)} \end{array}$$ [5]

$$\begin{array}{rl} & \text{PH-OOH}+\text{RCHO (aldehyde)}\\ & \;\to \text{RCOOH}+{}^{*}\text{PH-OH (electrically excited}\\ & \;\text{hydroxyl compound)}\\ & {}^{*}\text{PH-OH}\to \text{P}+\text{H}{}_{2}\text{O}+hv\text{ (CL)} \end{array}$$ [6]

**Figure 15.**
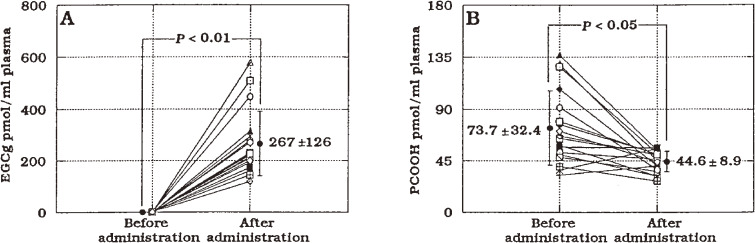
Plasma epigallocatechin gallate (EGCg) (A) and phosphatidylcholine hydroperoxide (PCOOH) (B) before tea catechin administration and 60 min after administration in humans. Values represent the mean ± SD of 18 subjects. Green tea extract (equivalent to 254 mg catechin) was administrated after 12 h of fasting. Adapted with permission from Ref. 88. Copyright 1999 American Chemical Society.

This technique enabled the testing of direct incorporation of dietary tea catechin into rat intestinal mucosal cells and its spectrometric confirmation. The emission spectrum (Emax 630 nm) of small intestinal mucosal cells prepared from rats after oral administration of epigallocatechin gallate (EGCg), a major green tea polyphenol, 23 mg/100 g body weight, was identical to the emission spectrum of authentic EGCg. This demonstrated that orally ingested EGCg is directly incorporated into intestinal mucosal cells. This is important evidence for the potent antioxidant actions of green tea EGCg in preventing atherosclerosis development. Next, CL-HPLC for the highly specific determination of the tea catechin EGCg, present in rat and human plasma, was developed.^87)^ The CL-HPLC system consisted of reversed-phase HPLC and a CL detector, where separated EGCg generates CL in a successive reaction post-column with the following two CL cocktails: 8.2 M acetaldehyde in 50 mM phosphate buffer (pH 7.4, containing 108 mg horseradish peroxidase/L) and 8.8 M hydrogen peroxide aqueous solution. Plasma EGCg was extracted using methanol. This method enabled the selective detection of free form EGCg at concentrations as low as 2 pmol, with 84% recovery. The EGCg concentration in fasted rat plasma was initially below the detection limit (<2 pmol/mL), but it increased to a maximum level (2284 pmol/mL plasma, 1047 ng/mL; calculated as 0.012% of ingested EGCg) at 30 min after a single oral supplementation of 50 mg EGCg per rat. The EGCg concentration in fasted human plasma was also initially below the detection limit and increased to 341 pmol/mL (156 ng/mL; calculated as 0.32% of ingested EGCg) at 60 min after a single oral intake of 97 mg EGCg per subject. The results indicated that tea catechin, EGCg, is absorbed from the digestive tract into the body in rats and humans, and that the CL-HPLC method established here is a powerful tool for studying the metabolic fate and bioavailability of food polyphenols. We also found that tea catechin supplementation increased antioxidant capacity and prevented phospholipid hydroperoxidation in human plasma.^88)^ Eighteen healthy male volunteers who orally ingested a green tea extract (254 mg of total catechins/subject) showed 267 pmol EGCg/mL of plasma at 60 min after administration. Plasma PCOOH levels correlated inversely with the increase in plasma EGCg level (Fig. 15).^88)^ The results suggested that drinking green tea contributes to preventing atherosclerosis and cardiovascular disease by increasing plasma antioxidant capacity in humans. The results of this study showed that longevity in Japan is promoted by the antioxidant action of green tea, consumed with meals to prevent atherosclerosis and myocardial infarction.

## 7 LC-MS/MS determination of PCOOH diastereomers, discrimination between enzymatic and auto-oxidation of PC, and oxidation mechanisms of TG and edible oils

**Figure 16.**
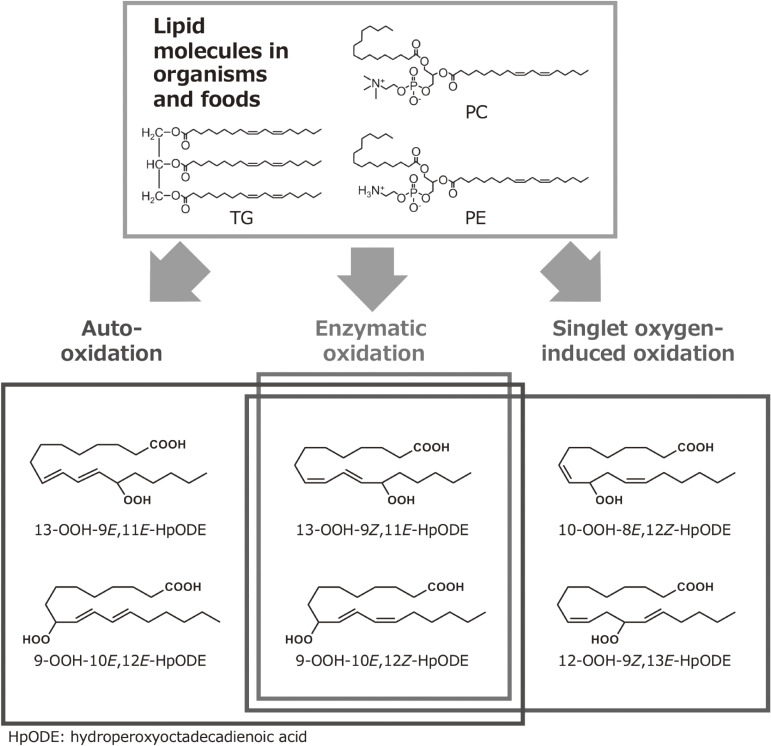
Lipid oxidation mechanism and isomeric structure. Lipid oxidation mechanism (radical, singlet oxygen, or enzymatic) can be estimated by analyzing the structures around the hydroperoxyl group.

In order to understand the mechanism of lipid oxidation of food and biological samples, it was necessary to precisely analyze peroxide products at the isomer level. The CL-HPLC method was able to analyze hydroperoxides at the lipid class level, such as PCOOH, SQOOH, and TGOOH, but analysis of individual isomers was difficult, due to the limitations of the chromatographic column. Therefore, an LC-MS/MS instrument, for which remarkable progress in analytical accuracy has been made, was chosen. This precise analysis made it possible to elucidate the mechanism of lipid peroxidation and to plan its preventive components (Fig. 16).

**Figure 17.**
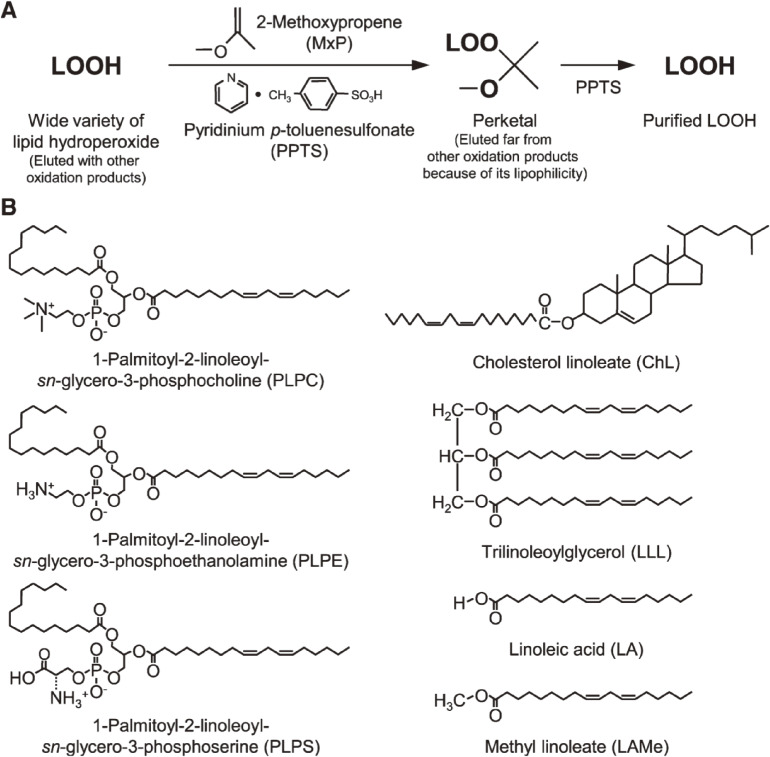
A: protocols investigated for the purification of lipid hydroperoxide (LOOH) using 2-methoxypropene (MxP). B: chemical structures of lipids [1-palmitoyl-2-linoleoyl-*sn*-glycero-3-phosphocholine (PLPC), 1-palmitoyl-2-linoleoyl-*sn*-glycero-3-phosphoethanolamine (PLPE), 1-palmitoyl-2-linoleoyl-*sn*-glycero-3-phosphoserine (PLPS), cholesteryl linoleate (ChL), trilinoleoylglycerol (LLL), linoleic acid (LA), and methyl linoleate (LAMe)]. Adapted with permission from Ref. 94. Copyright 2008 American Society for Biochemistry and Molecular Biology.

**Figure 18.**
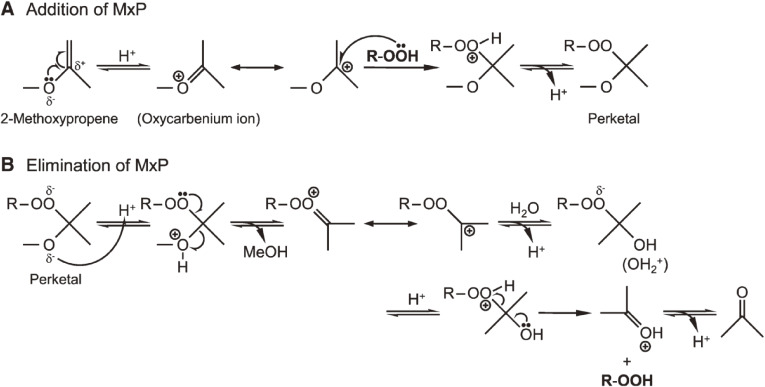
Presumed mechanism of the reaction between 2-methoxypropene (MxP) and hydroperoxide. (A): addition of MxP by nucleophilic addition of hydroperoxide to 2-methoxypropene. (B): elimination of MxP and regeneration of hydroperoxide. Adapted with permission from Ref. 94. Copyright 2008 American Society for Biochemistry and Molecular Biology.

First, a method for the preparation of a high-purity, stable lipid hydroperoxide (LOOH) standards for LC-MS/MS analysis was confirmed. A few previous studies^89–93)^ have reported that certain vinyl ether compounds [*i.e.*, 2-methoxypropene (MxP)] react with organic hydroperoxide to yield perketal for the preparation of pure LOOH. Because the reactivity of vinyl ethers with a LOOH other than fatty acid hydroperoxides has never been reported, we carried out reactions for the preparation of a wide variety of pure LOOHs. A phospholipid, cholesteryl ester, TG, or fatty acid were photo- or enzymatically oxidized, and the resultant crude sample containing hydroperoxide was reacted with MxP (Fig. 17, Table 5).^94)^ LC and MS confirmed that MxP selectively reacts with LOOH, yielding a stable MxP adduct (perketal) (Table 6). The lipophilic perketal was eluted at a position away from intact LOOH, identified, and isolated by LC. Upon treatment with acid, perketal released the original LOOH, which was finally purified by LC. Using this preparation procedure, for instance, we successfully produced 75 mg of pure PCOOH (>99%) from 100 mg of PC (Table 7).^94,95)^ According to our experience, about 9% of LOOH decomposed after 12 months during storage, even at −30 ℃. In contrast, LOOH-MxP (perketal) was more stable (about 97% preservation) than LOOH. Our method expanded the concept of the perketal method, which provides pure LOOH references (Fig. 18). The LOOHs prepared using the perketal method have been used as “gold standards” in LOOH methodology.

LC-MS/MS was employed to develop a method for the quantification of PCOOH molecular species (1-palmitoyl-2-hydroperoxy-octadecadienoyl-*sn*-glycero-3-phosphocholine, 16 : 0/HpODE PC), focusing on isomers such as 16 : 0/13-HpODE PC and 16 : 0/9-HpODE PC. Sodiated PCOOH ([M + Na]^+^, *m*/*z* 812), providing not only a known product ion (*m*/*z* 147), but also characteristic product ions (*m*/*z* 541 for 16 : 0/13-HpODE PC and *m*/*z* 388 for 16 : 0/9-HpODE PC). Thus, three MRMs can be performed. MRM (812/147) enables the determination of 16 : 0/HpODE PC, and MRM (812/541) and MRM (812/388) allows selective measurement of 16 : 0/13-HpODE PC and 16 : 0/9-HpODE PC, respectively (Table 8).^96,97)^ From this method, radical and/or enzymatic oxidation, rather than ^1^O_2_ oxidation, was confirmed to cause peroxidation of plasma PC in healthy subjects and patients with angiographically significant stenosis. These findings suggested the effect of dietary-derived antioxidants with radical scavenging and lipoxygenase (LOX)-inhibitory activity in the general inhibition of plasma lipid oxidation. In addition, ^1^O_2_ oxides of phospholipids were detected in areas of skin inflammation when sustained-release pharmaceuticals were implanted under the skin of rats. This was due to ^1^O_2_ produced by infiltrating macrophages, and the application of ^1^O_2_ scavengers or the intake of ^1^O_2_ quenchers, such as carotenoids, was effective in reducing this inflammation.

**Figure 19.**
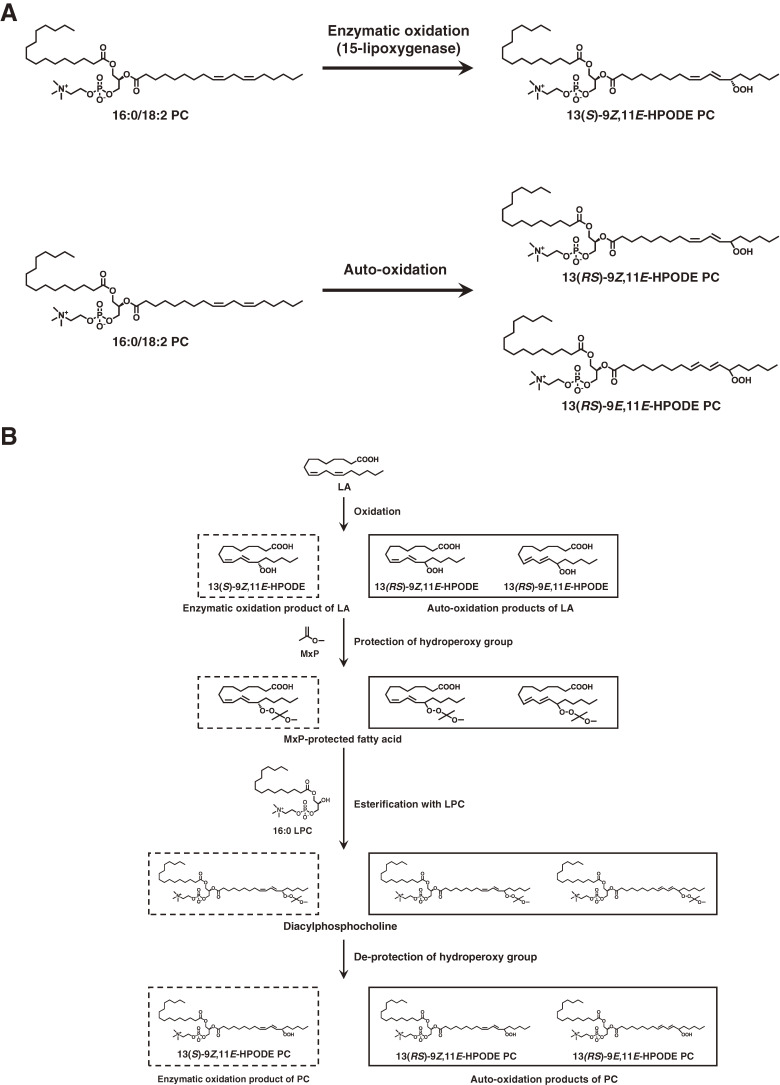
Chemical structures of phosphatidylcholine (PC) and phosphatidylcholine hydroperoxide (PCOOH). 13(*S*)-9*Z*,11*E*-HPODE PC is produced by enzymatic oxidation of PC (1-palmitoyl-2-linoleoyl-*sn*-glycero-3-phosphocholine (16 : 0/18 : 2 PC)). 13(*RS*)-9*Z*,11*E*-HPODE PC and 13(*RS*)-9*E*,11*E*-HPODE PC are formed by auto-oxidation of PC (A). The preparation scheme for the enzymatic oxidation product and the auto-oxidation products from LA is shown in (B). Adapted with permission from Ref. 99. Copyright 2016 Springer Nature.

**Figure 20.**
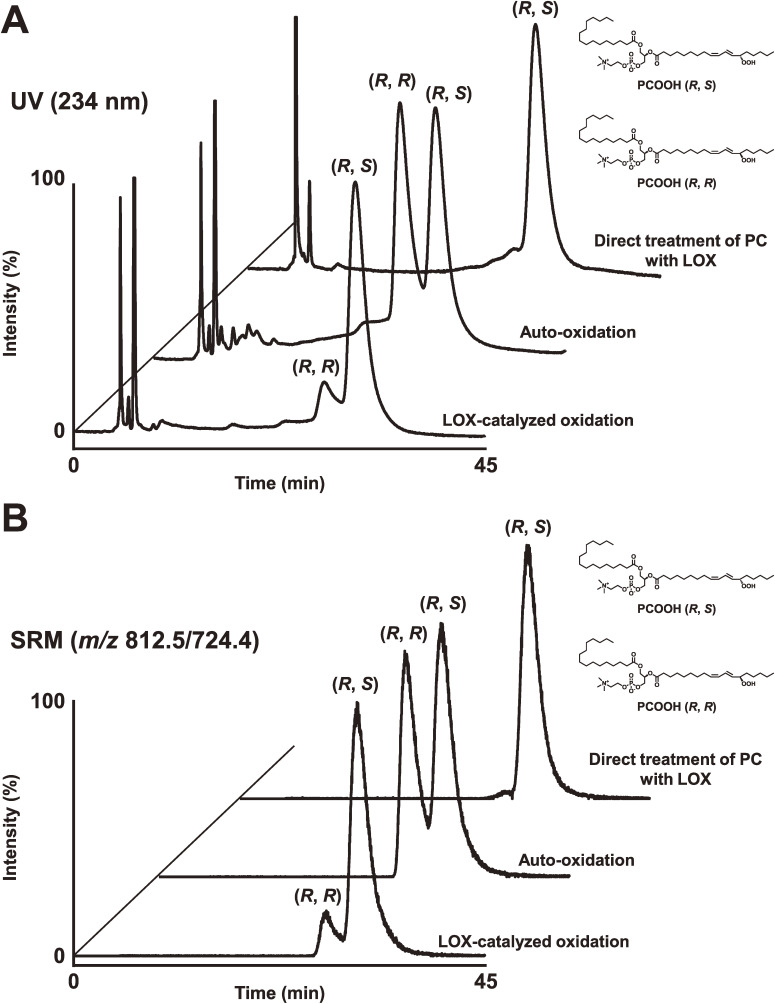
Diastereomer analysis of phosphatidylcholine hydroperoxide (PCOOH) bearing 13-9*Z*,11*E*-HPODE. PCOOH, prepared via lipoxygenase (LOX)-catalyzed oxidation or auto-oxidation, was subjected to stereoselective liquid chromatography ultraviolet mass spectrometry (LC-UV-MS) to evaluate the *R*/*S* configuration of the hydroperoxy group in the PCOOH molecule. PCOOH was detected by UV (234 nm; A) and structure-selective selected reaction monitoring (*m*/*z* 812.5/724.4; B). In addition, we prepared PCOOH by directly treating phosphatidylcholine (1-palmitoyl-2-linoleoyl-*sn*-glycero-3-phosphocholine, 16 : 0/18 : 2 PC) with LOX. The resultant PCOOH was analyzed by stereoselective LC-UV-MS. Adapted with permission from Ref. 98. Copyright 2015 Elsevier.

Enzymatic conversion of PC to PCOOH by LOX plays a crucial role in the biochemical processes that initiate phospholipid peroxidation *in vivo*. This was clarified by analysis of PCOOH bearing hydroperoxyl fatty acids with *S*-stereoconfiguration. Then, we synthesized PCOOH bearing 13*S*-hydroperoxy-9*Z*,11*E*-octadecadienoic acid (13(*S*)-9*Z*,11*E*-HpODE) using LOX, linoleic acid, and lysophosphatidylcholine (Fig. 19).^98,99)^ PCOOH bearing racemic 13-9*Z*,11*E*-HpODE was also prepared. Liquid chromatography equipped with CHIRALPAK OP (+) (poly (o-pyridyl diphenylmethacrylate) coated on silica), a UV detector, and a quadrupole-time-of-flight mass spectrometer (chiral stationary phase [CSP]-HPLC-UV-MS/MS) was employed, achieving diastereomer separation of PCOOH stereoisomers with excellent resolution and peak shape (Fig. 20).^98)^ This was the first study reporting the diastereomer separation of PCOOH.

**Figure 21.**
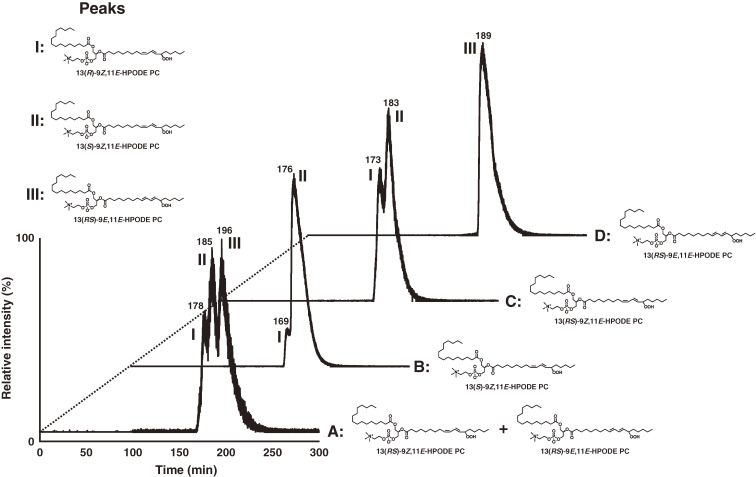
Diastereomer and/or *cis*-*trans* separation of the mixture of 13(*RS*)-9*Z*,11*E*-HPODE PC and 13(*RS*)-9*E*,11*E*-HPODE PC (chromatogram A), 13(*S*)-9*Z*,11*E*-HPODE PC (chromatogram B), 13(*RS*)-9*Z*,11*E*-HPODE PC (chromatogram C), and 13(*RS*)-9*E*,11*E*-HPODE PC (chromatogram D) using a combination of CHIRALPAK OP (+) and IB-3 columns. PCOOH isomers were analyzed by chiral stationary phase high performance liquid chromatography–tandem mass spectrometry (CSP-HPLC-MS/MS) with structure-selective SRM (*m*/*z* 812/541), and peaks were identified as follows: peak I, 13(*R*)-9*Z*,11*E*-HPODE PC; peak II, 13(*S*)-9*Z*,11*E*-HPODE PC; peak III, 13(*RS*)-9*E*,11*E*-HPODE PC. Adapted with permission from Ref. 99. Copyright 2016 Springer Nature.

**Figure 22.**
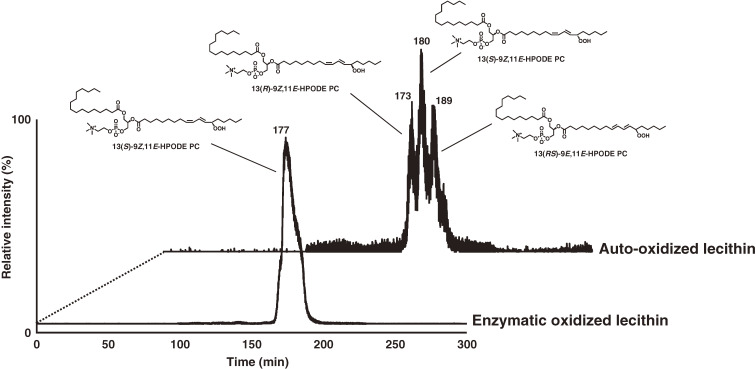
Distinction between enzymatic oxidation and auto-oxidation of an actual sample (oxidized lecithin). Oxidized lecithin samples were analyzed by chiral stationary phase high performance liquid chromatography–tandem mass spectrometry (CSP-HPLC-MS/MS) equipped with CHIRALPAK OP (+) and CHIRALPAK IB-3 columns. PCOOH isomers were detected by structure-selective SRM (*m*/*z* 812/541). Adapted with permission from Ref. 99. Copyright 2016 Springer Nature.

Because enzymatic oxidation progresses concurrently with auto-oxidation, we need to distinguish them further to elucidate the role of PC peroxidation in disease pathogenesis and in food deterioration. Thus, we synthesized the enzymatic oxidation product 13(*S*)-9*Z*,11*E*-HpODE PC and the auto-oxidation products 13(*RS*)-9*Z*,11*E*-HpODE PC and 13(*RS*)-9*E*,11*E*-HpODE PC, which are used as standards in CSP-HPLC-UV-MS/MS analysis. The CHIRALPAK OP (+) column separated 13(*R*)-9*Z*,11*E*-HpODE PC and 13(*S*)-9*Z*,11*E*-HpODE PC, and CHIRALPAK IB-3 separated 13(*S*)-9*Z*,11*E*-HpODE PC and 13(*RS*)-9*E*,11*E*-HpODE PC (Fig. 21). The established method, CSP-HPLC-UV-MS/MS, achieves stereoselective and *cis-trans* separation of PCOOH, and it is useful for distinguishing enzymatic oxidation and auto-oxidation reactions occurring *in vivo* and in foods (Fig. 22).^99)^

Characteristic lipid hydroperoxide isomers are formed by different oxidation mechanisms, *i.e.*, photo-oxidation or auto-oxidation. For example, linoleic acid is photo-oxidized to 13-9*Z*,11*E*-HpODE, 12-9*Z*,13*E*-HpODE, 10-8*E*,12*Z*-HpODE, whereas 13-9*Z*,11*E*-HpODE, 13-9*E*,11*E*-HpODE, 9-10*E*,12*Z*-HpODE, and 9-10*E*,12*E*-HpODE are formed by auto-oxidation.^100)^ Therefore, oxidation mechanisms can be evaluated by analyzing these characteristic positional and *cis*/*trans* lipid hydroperoxide isomers with CSP-HPLC-UV-MS/MS.^101)^ Soybean oil, rice bran oil, and olive oil were first subjected to light exposure or heating and were treated with Lipase AY-30, which hydrolyses the ester bonds in TG regardless of the fatty acid position or chain length. Therefore, all TGs were hydrolyzed into a crude sample containing glycerol and fatty acids, including HpODE isomers. To the best of our knowledge, this was the first study applying lipase to analyze HpODE isomers from oxidized edible oils. Lipase is also useful for the analysis of other complex lipid hydroperoxides, *e.g.*, phospholipid hydroperoxides, TG hydroperoxides, and cholesterol hydroperoxides. HpODE isomers were detected in soybean oil and olive oil, despite being analyzed immediately after opening. The HpODE isomers that are mainly detected even in unoxidized oils were the isomers characteristic of photo-oxidation, *e.g.*, 13-9*Z*,11*E*-HpODE, 12-9*Z*,13*E*-HpODE, 10-8*E*,12*Z*-HpODE, and 9-10*E*,12*Z*-HpODE. This indicated that photo-oxidation had already occurred in edible oils before opening, *e.g.*, during the manufacturing process, transportation, and/or sale. In light-exposed edible oils, these isomers specific to photo-oxidation were found in larger amounts compared with oils that were not exposed to light. Of note, light-exposed edible oils contained small amounts of 13-9*E*,11*E*-HpODE and 9-10*E*,12*E*-HpODE, which are isomers that result from the auto-oxidation of linoleic acid. This indicated that the lipid hydroperoxides generated by photo-oxidation became lipid peroxyl radicals, which are free radicals that can initiate the auto-oxidation (radical oxidation) of other lipids. The results implied that light illumination, *e.g.*, during sale and kitchen storage, can induce photo-oxidation, as well as a small degree of auto-oxidation of edible oils. On the other hand, in edible oils subjected to heating, HpODE isomers characteristic of auto-oxidation, *i.e.*, 13-9*E*,11*E*-HpODE and 9-10*E*,12*E*-HpODE, were largely increased. HpODE isomers specific to photo-oxidation, 12-9*Z*,13*E*-HpODE and 10-8*E*,12*Z*-HpODE, were also detected in heated edible oils, but, considering their low concentrations, these isomers were presumably present before the oils were heated.^101)^ These results indicated that the heating of edible oils, *e.g.*, during cooking, may induce the auto-oxidation of edible oils.

Soybean oil and rice bran oil contained about 100 mg/100 g of vitamin E homologues, which are well-known lipophilic antioxidants in foods. Vitamin E is particularly effective in preventing auto-oxidation.^102)^ A characteristic of rice bran oil is the fact it contains not only tocopherols but also tocotrienols.^103–105)^ Tocotrienols are known to demonstrate similar antioxidant activities as tocopherols; therefore, the amount of HpODE isomers characteristic of auto-oxidation, such as 13-9*E*,11*E*-HpODE and 9-10*E*,12*E*-HpODE, did not differ compared with soybean oil.^101)^ On the other hand, olive oil contained lower levels of vitamin E homologues than soybean oil and rice bran oil. This was reflected in the ratio of 13-9*Z*,11*E*-HpODE to 13-9*E*,11*E*-HpODE and 9-10*E*,12*Z*-HpODE to 9-10*E*,12*E*-HpODE, where the ratios were distinctly different between light-exposed soybean oil and olive oil. Because 13-9*E*,11*E*-HpODE and 9-10*E*,12*E*-HpODE are isomers characteristic of auto-oxidation, high contents of these isomers indicate that auto-oxidation occurred at higher rates in light-exposed olive oil, compared with soybean oil and rice bran oil. The results indicated that the auto-oxidation of edible oils was affected by the amount of vitamin E homologues, rather than the composition of vitamin E homologues. In addition, it is worth noting that HpODE isomers were detected in each olive oil sample despite containing only 6% linoleic acid. This content was significantly lower than in soybean oil and rice bran oil (53% and 35% respectively). However, the fact that olive oil contained similar HpODE amounts compared with soybean oil and rice bran oil indicated that olive oil was more susceptible to lipid oxidation than soybean oil and rice bran oil.^101)^ A cause of this lipid oxidation may be the natural components in olive oil, which may act as photo-sensitizers, such as chlorophyll derivatives that induce photo-oxidation. The results suggested, for the first time, that the oxidation mechanisms of lipids and edible oils can be evaluated by analyzing the characteristic positional and *cis*/*trans* isomers of lipid hydroperoxides, *i.e.*, HpODE isomers. With our research, the combination of the CSP-HPLC-UV-MS/MS method and lipase was proven useful for the evaluation of lipid oxidation mechanisms of foods and biological systems.

**Figure 23.**
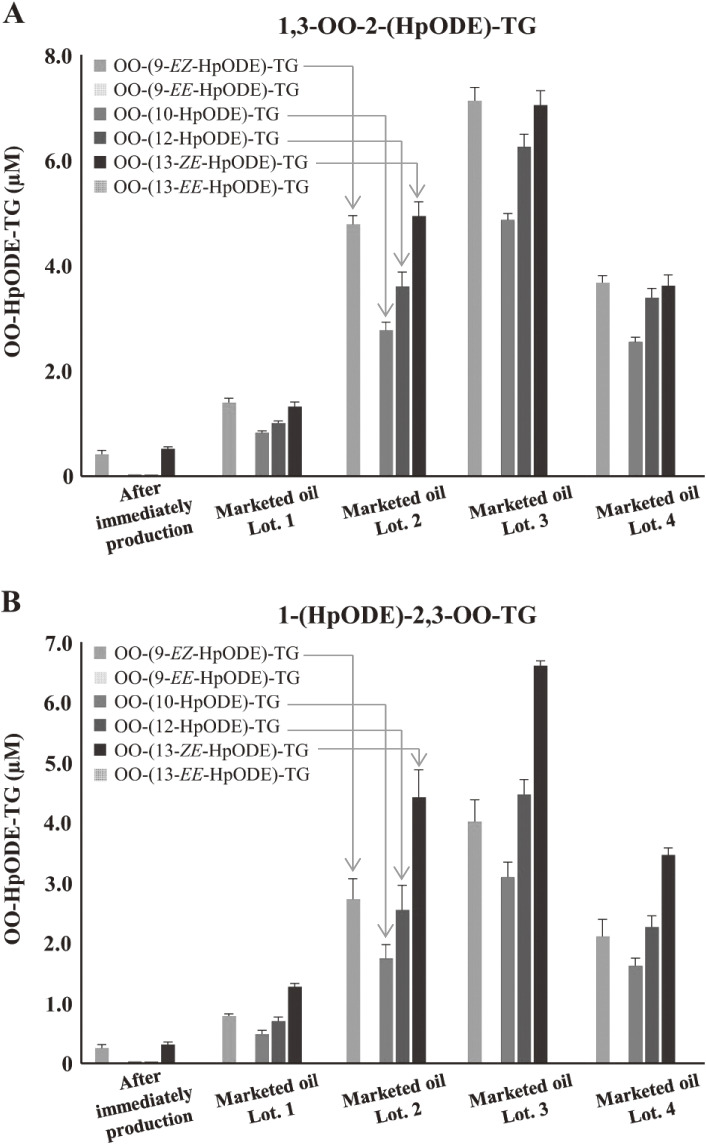
Concentrations of OO-HpODE-TG isomers in marketed canola oil (A and B). Marketed oils were analyzed immediately after opening. Oils were diluted 100-fold with hexane, and a portion (50 µL) was analyzed using optimized liquid chromatography–tandem mass spectrometry (LC-MS/MS) multiple reaction monitoring (MRM) conditions. Adapted from Ref. 106.

TG, the main component of edible oil, is oxidized by thermal- or photo-oxidation to form TGOOH as the primary oxidation product. Because TGOOH and its subsequent oxidation products cause the deterioration of oil quality and various toxicities, preventing the oxidation of edible oils is essential. Therefore, understanding the oxidation mechanisms that cause the formation of TGOOH is necessary. Isomeric information on lipid hydroperoxide provides insights on oil oxidation mechanisms; therefore, we focused on dioleoyl-(hydroperoxyl octadecadienoyl)-TG (OO-HpODE-TG) isomers, which are the primary oxidation products of the most abundant TG molecular species (dioleoyl-linoleoyl-TG) in canola oil.^106)^ To secure highly selective and sensitive analysis, authentic OO-HpODE-TG isomer references such as hydroperoxide positional/geometrical isomers were synthesized and analyzed with HPLC-MS/MS. With the use of this method, photo- or thermal-oxidized edible oils were analyzed. Although dioleoyl-(10-hydroperoxy-8*E*,12*Z*-octadecadienoyl)-TG (OO-(10-HpODE)-TG) and dioleoyl-(12-hydroperoxy-9*Z*,13*E*-octadecadienoyl)-TG (OO-(12-HpODE)-TG) were characteristically detected in photo-oxidized oils, dioleoyl-(9-hydroperoxy-10*E*,12*E*-octadecadienoyl)-TG and dioleoyl-(13-hydroperoxy-9*E*,11*E*-octadecadienoyl)-TG were found to increase depending on temperature in thermal-oxidized oils (Fig. 23). The results demonstrated that our methods to evaluate oil oxidation in levels were unquantifiable with peroxide values and allowed for the determination of oil oxidation mechanisms.^106)^ From the analysis of marketed canola oils, photo-oxidized products, *i.e.*, OO-(10-HpODE)-TG and OO-(12-ODE)-TG were characteristically accumulated, compared with the oil analyzed immediately after production. The method we described is valuable in the understanding of oil and food oxidation mechanisms and may be applied to the development of preventive methods against food deterioration.

## 8 Membrane phospholipid hydroperoxides in Alzheimer’s disease (AD) patients, in a primate model of subarachnoid hemorrhage, and in a mouse model for hepatitis C virus-associated hepatocarcinogenesis

**Figure 24.**
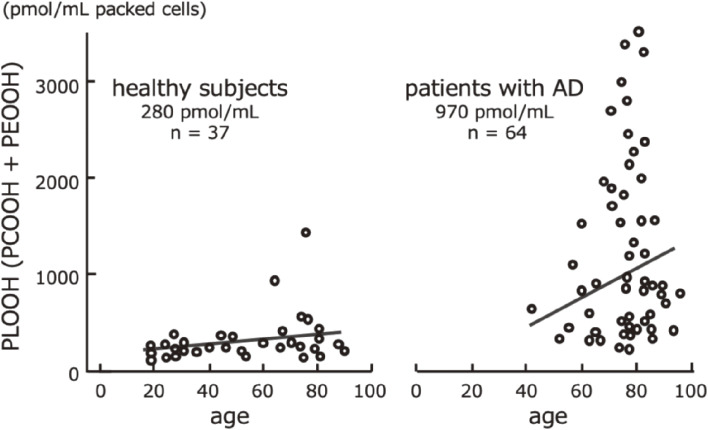
Phospholipid hydroperoxide (PLOOH) content of the erythrocyte membranes in healthy subjects and patients with Alzheimer’s disease (AD). Adapted from Ref. 107.

In 1992, using the CL-HPLC method, Miyazawa *et al.* first reported the significant accumulation of phospholipid hydroperoxides (PCOOH and PEOOH) in red blood cell (RBC) membranes in AD patients, compared with normal subjects (Fig. 24).^107)^ Accumulation of phospholipid peroxide in RBCs appears to reduce the ease of capillary transit and the ability to supply oxygen to peripheral tissues. We noted for the first time in the world that significantly higher concentrations of PCOOH and PEOOH accumulated in the RBCs of patients with AD than in healthy RBCs, which may be involved in the progression of the disease, including reduced oxygen supply to the brain. Even in healthy individuals, circulating RBCs with high levels of PCOOH and PEOOH in the lipid membrane increase with physiological aging, but the concentrations are not as high as in patients with AD.^108)^ These studies also showed that in the elderly and people with AD, the ability to remove aged RBC and erythropoiesis was impaired.

We hypothesized that amyloid β-peptide (Aβ) peroxidizes RBC lipid membranes by oxidative impairment of their capacity to deliver oxygen to the brain. These processes are implicated in the pathogenesis of AD. Although plasma Aβ has been investigated thoroughly, the distribution of Aβ in human RBCs was still unclear. We quantitated Aβ40 and Aβ42 in human RBCs using ELISA assays and provided evidence that significant amounts of Aβ can be detected in RBCs and that RBC Aβ levels increased with aging.^109)^ On the other hand, administering an antioxidant supplement (astaxanthin, a polar carotenoid) in humans was found to decrease RBC Aβ and oxidative stress marker levels.^110)^ These results suggested that plasma Aβ40 and Aβ42 bind to RBCs (possibly with aging), implying a pathogenic role of RBC Aβ. Moreover, the data indicated that RBC Aβ40 and Aβ42 may constitute AD biomarkers. As a preventive strategy, therapeutic application of dietary polar carotenoids (*i.e.*, xanthophylls such as astaxanthin) as Aβ42-lowering agents in RBCs was considered as a possible anti-dementia therapeutic option.

**Figure 25.**
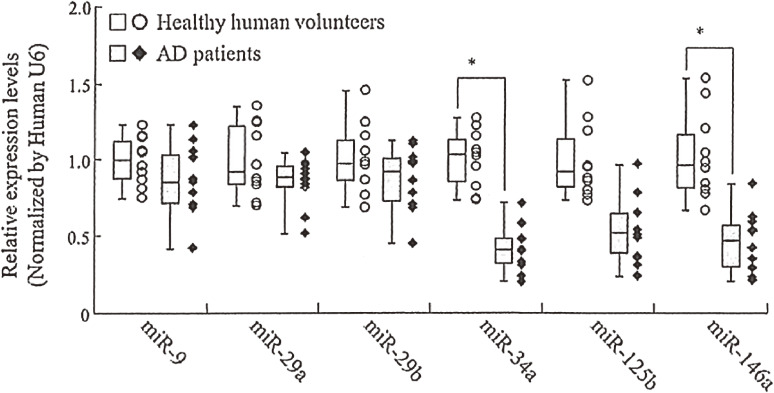
miRNA levels in plasma from healthy human volunteers and Alzheimer’s disease (AD) patients. Values are mean ± SD (*n* = 10). Differences were considered significant compared with the control subjects (*p < 0.05). Adapted with permission from Ref. 111. Copyright 2014 IOS Press.

The development of AD biomarkers, other than oxidized RBCs, remains a challenge. MicroRNA (miRNA/miR) profiling of biological fluids has been suggested as a diagnostic tool for several pathologic conditions. We measured six candidate miRNAs (miR-9, miR-29a, miR-29b, miR-34a, miR-125b, and miR-146a) in plasma and cerebrospinal fluid (CSF) of AD and normal subjects using quantitative reverse transcriptase-polymerase chain reaction (qRT-PCR) to evaluate their potential usability as AD biomarkers (Table 9).^111)^ The qRT-PCR results showed that plasma miR-34a and miR-146a levels, and CSF miR-34a, miR-125b, and miR-146a levels in AD patients were significantly lower than in control subjects (Fig. 25). On the other hand, CSF miR-29a and miR-29b levels were significantly higher than in control subjects. These data provide a possibility that miRNAs detected in plasma and CSF can serve as biomarkers for AD.

Aside from the accumulation of Aβ peptide in the brain, AD may be associated with the peroxidation of major phospholipids, *e.g.*, PC, and degradation of antioxidative phospholipids, *e.g.*, ethanolamine plasmalogen (PlsEtn).^112–114)^ In addition to its presence in the brain, Aβ is also found in blood; however, information about the levels of PCOOH and PlsEtn in the blood of patients with AD is limited. By assuming a possible interaction between Aβ, PCOOH, and PlsEtn in the blood circulation, we evaluated the levels of these molecules and correlations in blood samples that had been obtained from our study on PCOOH measurement. Then, we found that compared with the controls, plasma from patients with AD showed lower concentrations of PlsEtn species, especially PlsEtn bearing a docosahexaenoic acid (DHA) moiety.^115)^ In addition, lower PlsEtn and higher PCOOH levels were observed in the RBCs of AD patients (Table 10). In both AD and control blood samples, RBC PCOOH levels tended to correlate with Aβ40 plasma levels, and each PlsEtn species showed different correlations with plasma Aβ. These results, together with *in vitro* data suggesting that Aβ aggregation is due to a decrease in PlsEtn levels with DHA, led us to deduce that Aβ alters the levels of PCOOH and PlsEtn species observed in the blood of patients with AD.^115)^

Our study showed that the RBCs of AD patients are in an excessively oxidized state, *i.e.*, with a high accumulation of PCOOH. Then, we further evaluated the antioxidant carotenoid composition and PLOOH (PCOOH and PEOOH) concentrations in 28 normal control subjects (average age 74 years) and 28 patients with AD (average age 72 years).^116)^ Lutein was found to be a predominant RBC antioxidant carotenoid, and its concentration in AD patients was significantly lower than in control subjects. An inverse relationship was typically confirmed between RBC lutein and PLOOH concentrations in AD patients. This suggested that RBC lutein contributes to prevent RBC membrane lipid oxidation, which is characteristic of AD patients. Food-grade lutein (9 mg/day/2 or 4 weeks) intake resulted in a decrease in PLOOH concentration in RBCs, even in normal subjects.^117)^ Using the unicellular dietary green algae chlorella, *Chlorella pyrenoidosa*, which is rich in lutein,^118)^ we conducted a randomized, double-blind, placebo-controlled human trial to assess the impact of chlorella ingestion (8 g chlorella/day/person; equivalent to 22 mg lutein/day/person) on lutein concentrations in the RBCs and plasma of normal senior subjects for a total of 2 months.^119)^ After 1 or 2 months of ingestion, RBC and plasma lutein concentrations increased in the *Chlorella*-ingested group. RBC PLOOH concentrations were lower than the concentrations before supplementation after a total of 2 months of ingestion. The results indicate that dietary *Chlorella* improves RBC antioxidant status and prevents membrane phospholipid peroxidation in senescent RBCs.

Tirilazad mesylate has been used in an attempt to prevent cerebral vasospasm after subarachnoid hemorrhage (SAH), although the actual targets of this agent *in vivo* have thus far been controversial. A cooperative study suggested that tirilazad might have a neuroprotective effect in patients with SAH.^120–122)^ However, its precise mechanism of action in improving vasospasms has yet to be fully understood, especially for oxidative stress on membrane phospholipids. Then, we tried to measure PCOOH and PEOOH levels using the CL-HPLC method in a primate model of SAH to determine the effects of tirilazad on vasospasms.^123)^ Tirilazad was provided by the Upjohn Company (Kalamazoo, Mich.). Fourteen *Macaca* monkeys of both sexes were randomly assigned into 2 groups: a the tirilazad group receiving a dosage of 0.3 mg/kg and a placebo group receiving only the vehicle in which tirilazad was delivered. After the induction of experimental SAH around the right middle cerebral artery on day 0, tirilazad or vehicle were administered intravenously every 8 h for 6 days. On day 7, the animals were sacrificed after angiography and regional cerebral blood flow measurements were performed. The levels of PCOOH and PEOOH were measured in the clots, bilateral parietal cortices, right frontal cortex contact with clots, cerebellar hemispheres, bilateral middle cerebral arteries, and basilar arteries. In the placebo group, a significant vasospasm occurred in the cerebral arteries on both sides, but most prominently on the right side. The degree of vasospasm in the cerebral arteries was significantly attenuated in the tirilazad group. There were no significant differences in regional cerebral blood flow, PCOOH, and PEOOH levels in the clots, cerebral cortices, and cerebellar hemispheres between the two groups. In contrast, the levels of PCOOH in the cerebral arteries were significantly higher in the placebo group than in the tirilazad group (*P* < 0.025). Remarkably, the tirilazad treatments eliminated PCOOH in all vascular territories after SAH. These findings indicated that PCOOH in the artery wall is an important indicator of vasospasms, and the inhibition of PCOOH explained the efficacy of tirilazad on vasospasm in a primate model.

**Figure 26.**
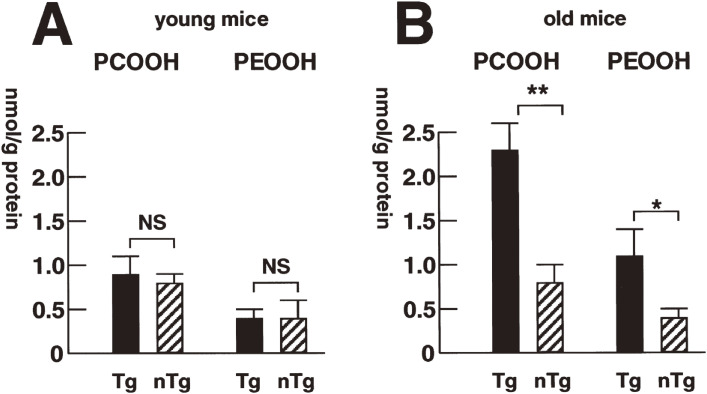
Levels of lipid peroxidation in core genes of transgenic and control mice. (A): young mice aged 3–12 months. (B): old mice >16 months of age. The hydroperoxide products of phosphatidylcholine hydroperoxide (PCOOH) or phosphatidylethanolamine hydroperoxide (PEOOH) were determined in liver tissue homogenates of transgenic mice (Tg) and nontransgenic control mice (nTg). The data are the means ± SE (n = 5 in each group). NS, not statistically significant. *, P < 0.05; **, P < 0.01. Adapted with permission from Ref. 128. Copyright 2001 American Association for Cancer Research.

The mechanism of hepatocarcinogenesis in hepatitis C virus (HCV) infection is still undefined. One possibility is the involvement of oxidative stress, which can produce genetic mutations as well as gross chromosomal alterations and contribute to cancer development. It was shown that after a long period, the core protein of HCV induces hepatocellular carcinoma (HCC) in transgenic mice with marked hepatic steatosis but without inflammation, indicating a direct involvement of HCV in hepatocarcinogenesis.^124–127)^ To elucidate the biochemical events before the development of HCC, we examined several parameters of oxidative stress and redox homeostasis in a mouse model of HCV-associated HCC.^128)^ For young mice aged 3–12 months, there was no significant difference in the levels of PCOOH and PEOOH in liver tissue homogenates between transgenic and non-transgenic control mice. In contrast, PCOOH levels were increased by 180% in old core gene transgenic mice >16 months old (Fig. 26). Concurrently, there was a significant increase in catalase activity, and the levels of total and reduced glutathione decreased in the same mice. A direct *in situ* determination by CL revealed an increase in hydroperoxide products by 170% even in young transgenic mice, suggesting that hydroperoxides were overproduced but immediately removed by an activated scavenger system in young mice. Electron microscopy revealed lipofuscin granules, secondary lysosomes carrying various cytoplasmic organelles, and disruption of the double membrane structure of mitochondria, and PCR analysis disclosed a deletion in mitochondrial DNA. Notably, alcohol caused a marked increase in PCOOH levels in transgenic mice, suggesting synergism between alcohol and HCV in hepatocarcinogenesis. The HCV core protein thus alters the oxidant/antioxidant state in the liver in the absence of inflammation and may thereby contribute to, or facilitate, at least in part, the development of HCC in HCV infection.

## 9 Conclusion

**Figure 27.**
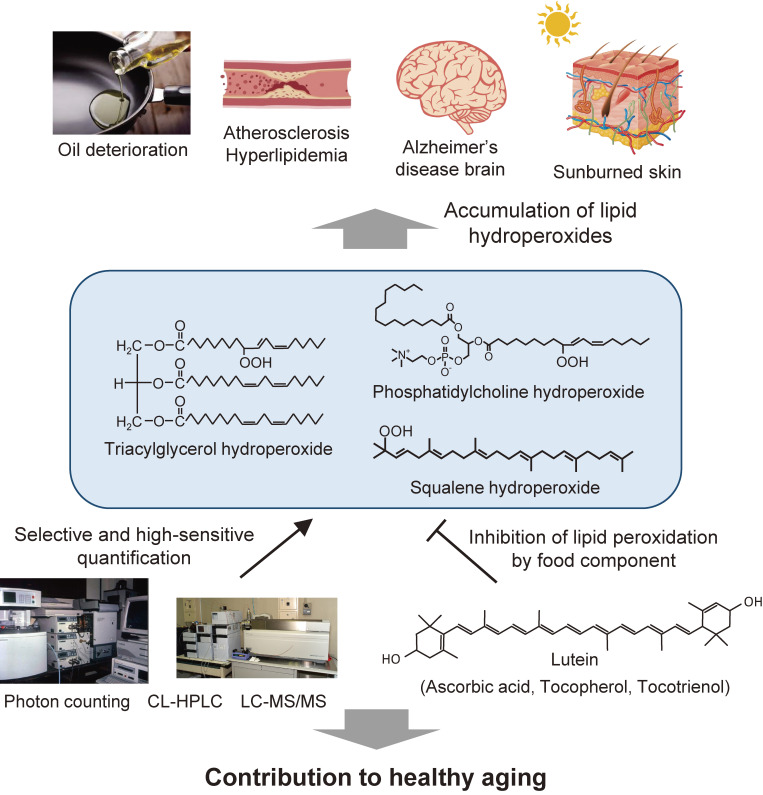
Research summary for lipid hydroperoxides in nutrition, health, and diseases.

Of the lipids, proteins, and carbohydrates that are the major constituents of food and living organisms, lipids are the most reactive with oxygen molecules. Demonstrating the production of lipid peroxides by oxidative stress is one of the most rewarding and challenging endeavors in food hygiene and biological health. Therefore, we pioneered and researched methods for the detection, quantification, and precise structural analysis of lipid hydroperoxides, which are the primary products of the peroxidation reaction, in order to demonstrate it. First, we developed the world’s most sensitive single photon counting device and applied it to elucidate the production of excited molecules associated with oil and fat degradation and radical reactions in biological tissues. In addition, such endeavors have stimulated the development of the CL-HPLC method for the detection and quantification of lipid hydroperoxides, specifically at the hydroperoxide group, which has been used to detect and quantify PCOOH, and the formation of SQOOH in sebum due to sunburn, which are related to cellular aging. PCOOH involvement was identified in the development of hyperlipidemia and atherosclerosis, which were inhibited by green tea consumption. Using both CL-HPLC and LC-MS/MS, we found that photo-oxides are formed in the very early stages of food oil degradation, and that radical oxides accumulate afterwards. The significance of utilizing ^1^O_2_ scavenging components such as carotenoids and other ^1^O_2_ scavengers in the prevention of edible oil degradation was highlighted. We also demonstrated H_2_O_2_ and ozonide formation in food processing and ozone disinfection. From these studies, we found high levels of aged RBCs with accumulated phospholipid peroxidation in dementia patients and showed that lipid peroxidation can be inhibited by lutein-rich chlorella intake. Furthermore, oxidative stress in the absence of inflammation in a mouse model for hepatitis C virus-associated hepatocarcinogenesis was clarified. Thus, the discovery of new phenomena in oxygen stress in food and in the human body revealed by our studies will strengthen the contribution of food as support for our body for health and longevity (Fig. 27).

## Figures and Tables

**Table 1.** Phosphatidylcholine hydroperoxide (PCOOH) and phosphatidylethanolamine hydroperoxide (PEOOH) concentrations in microsomes and plasma membranes of rat hepatocytes

	Age (months)	PCOOH (pmol/mg protein)	PEOOH (pmol/mg protein)	PCOOH + PEOOH (pmol/mg protein)
Microsomes	2 (*n* = 5)	12 ± 5	6 ± 3	17 ± 8
	17 (*n* = 9)	34 ± 14*	12 ± 5**	46 ± 18*
Plasma membranes	2 (*n* = 5)	23 ± 4	9 ± 2	31 ± 5
	17 (*n* = 9)	225 ± 96***	81 ± 31***	306 ± 126***

Values represent mean ± SD. Figures in parenthesis show the number of rats examined.

*P < 0.01, compared with PCOOH or PCOOH + PEOOH in the 2-month-old group; **P < 0.05, compared with PEOOH in the 2-month-old group; ***P < 0.001, compared with the respective hydroperoxide in the 2-month-old group.

Adapted with permission from Ref. 47. Copyright 1998 Elsevier.

**Table 2.** Determination of hydrogen peroxide found in coffee drinks by chemiluminescence detection-high performance liquid chromatography (CL-HPLC)

Coffee drink	H_2_O_2_ found (µM)
Normal	67.1 ± 3.8
Caffeine-free	64.7 ± 2.1
Espresso	99.7 ± 9.7
Drip	165.0 ± 5.3

Data represent means ± standard errors (*n* = 4). Normal, caffeine-free, and espresso coffee drinks were prepared by dissolving 2.0 g of powdered instant coffee in 140 mL of boiled water. Drip coffee was prepared by pouring 150 mL of boiling water onto 15.0 g of freshly milled coffee beans on a filter paper.

Adapted with permission from Ref. 51. Copyright 1994 Elsevier.

**Table 3.** Plasma phosphatidylcholine hydroperoxide (PCOOH) in hyperlipidemia

Subjects	*n*	PCOOH (nmol/L)^a^	*P*	
			*vs* controls	*vs* normalized
Controls	47	160 ± 65		
Hyperlipidemia				
Normalized	13	202 ± 17	NS^b^	
Type IIa	34	322 ± 32	<0.05	<0.01
Type IIb	24	353 ± 28	<0.01	<0.01
Type IV	23	397 ± 48	<0.01	<0.001

^a^Mean ± SE.

^b^NS, not significant.

Adapted with permission from Ref. 70. Copyright 2000 Oxford University Press.

**Table 4.** Maximum emission wavelength (Emax) of chemiluminescence from flavonoids in the presence of hydrogen peroxide, acetaldehyde, and horseradish peroxidase

Compound	Substituent position		Emax (nm)	Chemiluminescence intensity (×10^4^ counts/500 sec)
Catechins	(R1)	(R2)		990
Epigallocatechin-3-gallete	OH	Ga^a^	630	950
Epigallocatechin	OH		630	6
Epicatechin	H		630	
Theaflavins	(R3)	(R4)		
Theaflavin digallate	Ga	Ga	690	400
Theaflavin	H	H	690	24
Anthocyanins	(R5)	(R6)		
Delphinidin	OH	H	675	350
Cyanidin	H	H	675	95
Cyanidin-3-glucoside	H	Gluc^b^	675	156
Other phenolics				
Gallic acid			670	57
Caffeic acid			670	6
Curcumin			640	43
α-Tocopherol			No chemiluminescence	
(Ascorbic acid)			No chemiluminescence	

^a^Galloyl.

^b^Glucose.

The chemical structures are given below, and R1–R6 are substituted, as given above.
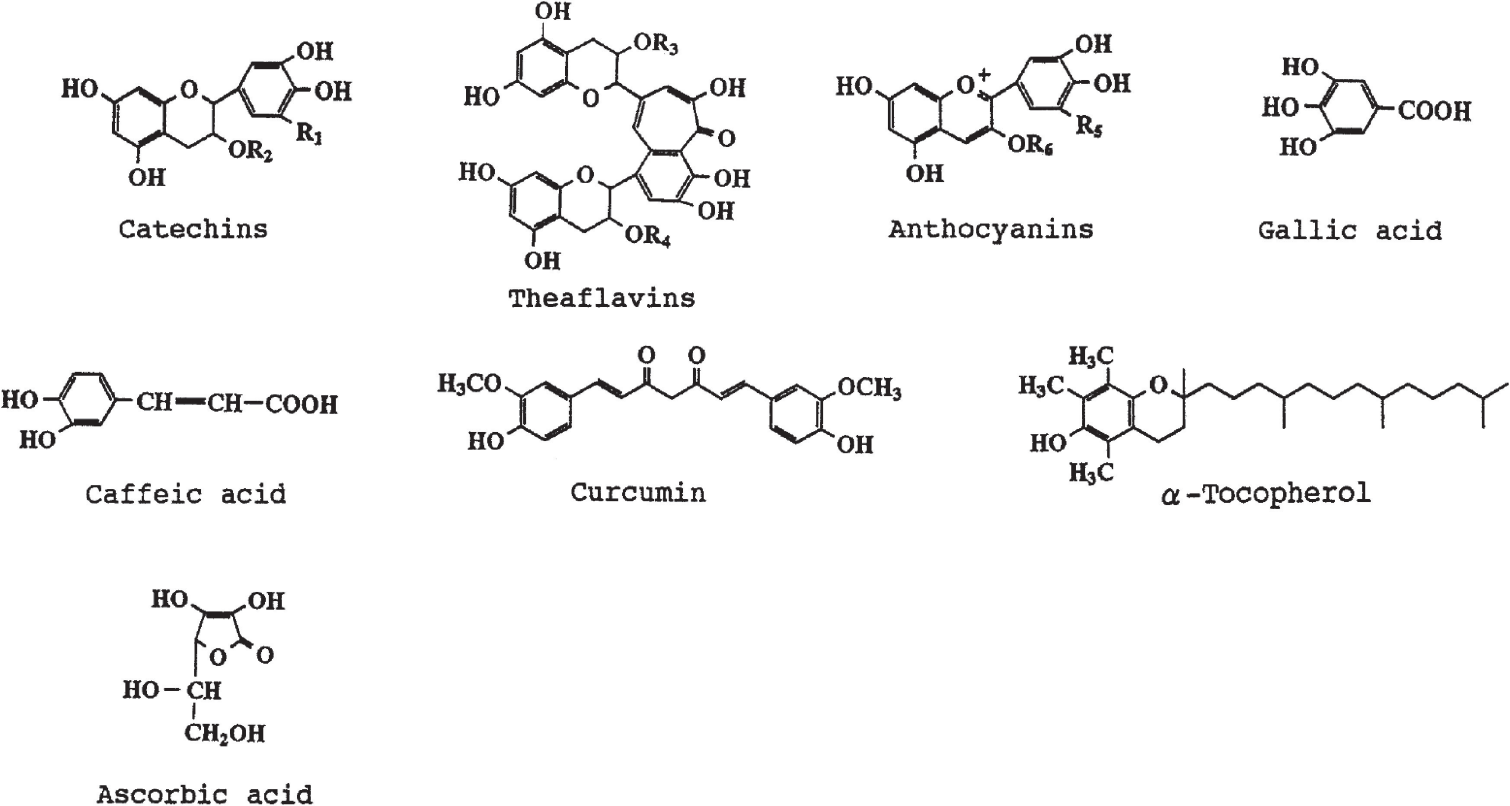

Adapted with permission from Ref. 86. Copyright 1998 Taylor & Francis.

**Table 5.** Optimal oxidation procedure to prepare lipid hydroperoxide (LOOH)

Lipid		Oxidation procedure	Time	Temp	Yielded LOOH^a^	
	mg		h	℃		mg
PLPC	100	RB-photo	8	4	PLPCOOH	30
	100	UV-photo	24	20	PLPCOOH	1.7
	100	LOX-1	12	40	PLPCOOH	96
PLPE	100	RB-photo	10	4	PLPEOOH	28
	100	UV-photo	24	20	PLPEOOH	1.4
	100	LOX-1	12	40	PLPEOOH	92
PLPS	100	RB-photo	10	4	PLPSOOH	30
	100	UV-photo	24	20	PLPSOOH	1.3
	100	LOX-1	12	40	PLPSOOH	94
ChL	100	RB-photo	8	4	ChLOOH	27
	100	UV-photo	24	20	ChLOOH	1.1
	100	LOX-1	24	40	ChLOOH	6.2
LLL	100	RB-photo	6	4	LLLOOH	31
	100	UV-photo	6	20	LLLOOH	35
	100	LOX-1	12	40	LLLOOH	9.4
LA	100	RB-photo	8	4	LAOOH	32
	100	UV-photo	12	20	LAOOH	35
	100	LOX-1	6	20	LAOOH	99
LAMe	100	RB-photo	8	4	LAMeOOH	34
	100	UV-photo	12	20	LAMeOOH	36
	100	LOX-1	8	20	LAMeOOH	97

LOX-1, lipoxygenase-1; PLPC, 1-palmitoyl-2-linoleoyl-*sn*-glycero-3-phosphocholine; PLPCOOH, 1-palmitoyl-2-hydroperoxyoctadecadienoyl-*sn*-glycero-3-phosphocholine; PLPE, 1-palmitoyl-2-linoleoyl-*sn*-glycero-3-phosphoethanolamine; RB, Rose Bengal; UV, ultraviolet.

^a^LC retention times and mass spectrometry (MS) profiles of hydroperoxides are as follows; PLPCOOH, 11.0 min, 790.6 [M + H]^+^; PLPEOOH, 10.5 min, 748.5 [M + H]^+^; PLPSOOH, 8.5 min, 790.4 [M − H]^−^; ChLOOH, 26.5 min, 703.6 [M + Na]^+^; LLLOOH, 23.0 min, 933.7 [M + Na]^+^; LAOOH, 15.5 min, 330.3 [M + NH_4_]^+^; LAMeOOH, 11.5 min, 349.3 [M + Na]^+^.

Adapted with permission from Ref. 94. Copyright 2008 American Society for Biochemistry and Molecular Biology.

**Table 6.** Optimal MxP reaction conditions to prepare perketal

Sample		Added PPTS	Added MxP		Total reaction volume	Time	Temp	Yielded perketal^c^		Perketal after LC isolation
in 20 mL of dichloromethane		mg/4 mL of dichloromethane	g	(mL)	mL	h	℃		mg	mg
PLPC	(RB-photo,^a^ 30 mg PLPCOOH^b^)	5	3	(4.0)	28	3	4	PLPCOOMxP	30	28
	(UV-photo, 1.7 mg PLPCOOH)	2	1	(1.3)	25	2	4	PLPCOOMxP	1.6	1.5
	(LOX-1, 96 mg PLPCOOH)	5	3	(4.0)	28	3	4	PLPCOOMxP	96	92
PLPE	(RB-photo, 28 mg PLPEOOH)	5	3	(4.0)	28	3	4	PLPEOOMxP	27	22
	(UV-photo, 1.4 mg PLPEOOH)	2	1	(1.3)	25	2	4	PLPEOOMxP	1.3	1.0
	(LOX-1, 92 mg PLPEOOH)	5	3	(4.0)	28	3	4	PLPEOOMxP	90	86
PLPS	(RB-photo, 30 mg PLPSOOH)	5	3	(4.0)	28	3	4	PLPSOOMxP	27	24
	(UV-photo, 1.3 mg PLPSOOH)	2	1	(1.3)	25	2	4	PLPSOOMxP	1.2	1.0
	(LOX-1, 94 mg PLPSOOH)	5	3	(4.0)	28	3	4	PLPSOOMxP	88	83
ChL	(RB-photo, 27 mg ChLOOH)	5	5	(6.7)	31	3	20	ChLOOMxP	26	20
	(UV-photo, 1.1 mg ChLOOH)	2	2	(2.7)	27	3	20	ChLOOMxP	1.0	0.9
	(LOX-1, 6.2 mg ChLOOH)	2	2	(2.7)	27	3	20	ChLOOMxP	5.9	5.3
LLL	(RB-photo, 31 mg LLLOOH)	10	5	(6.7)	31	3	20	LLLOOMxP	30	27
	(UV-photo, 35 mg LLLOOH)	10	5	(6.7)	31	3	20	LLLOOMxP	33	32
	(LOX-1, 9.4 mg LLLOOH)	5	3	(4.0)	28	3	20	LLLOOMxP	9.3	8.2
LA	(RB-photo, 32 mg LAOOH)	2	1	(1.3)	25	1	4	LAOOMxP	30	24
	(UV-photo, 35 mg LAOOH)	2	1	(1.3)	25	1	4	LAOOMxP	32	26
	(LOX-1, 99 mg LAOOH)	5	2	(2.7)	27	1	4	LAOOMxP	95	85
LAMe	(RB-photo, 34 mg LAMeOOH)	2	1	(1.3)	25	1	4	LAMeOOMxP	34	30
	(UV-photo, 36 mg LAMeOOH)	2	1	(1.3)	25	1	4	LAMeOOMxP	36	33
	(LOX-1, 97 mg LAMeOOH)	5	2	(2.7)	27	1	4	LAMeOOMxP	97	93

^a^Oxidation procedure.

^b^LOOH concentration in sample.

^c^LC retention times and MS profiles of perketals are as follows: PLPCOOMxP, 20.0 min, 862.6 [M + H]^+^; PLPEOOMxP, 18.5 min, 820.6 [M + NH_4_]^+^; PLPSOOMxP, 15.5 min, 862.5 [M − H]^−^; ChLOOMxP, 51.5 min, 775.6 [M + Na]^+^; LLLOOMxP, 45.0 min, 1005.8 [M + Na]^+^; LAOOMxP, 49.5 min, 402.3 [M + NH_4_]^+^; LAMeOOMxP, 33.5 min, 416.3 [M + NH_4_]^+^.

Adapted with permission from Ref. 94. Copyright 2008 American Society for Biochemistry and Molecular Biology.

**Table 7.** Optimal conditions for LOOH regeneration from perketal

Isolated perketal		Added PPTS	Total reaction volume	Time	Temp	Yielded LOOH^b^		LOOH after LC isolation	Purity of isolated LOOH
in 25 mL of chloroform/methanol (1 : 1)		mg/5 mL of chloroform/methanol (1 : 1)	mL	h	℃		mg	mg	%
PLPCOOMxP	(RB-photo,^a^ 28 mg)	10	30	6	4	PLPCOOH	27	16	98
	(UV-photo, 1.5 mg)	10	30	2	4	PLPCOOH	1.4	0.9	95
	(LOX-1, 92 mg)	10	30	12	4	PLPCOOH	90	75	>99
PLPEOOMxP	(RB-photo, 22 mg)	10	30	6	4	PLPEOOH	20	12	98
	(UV-photo, 1.0 mg)	10	30	2	4	PLPEOOH	0.9	0.6	94
	(LOX-1, 86 mg)	10	30	12	4	PLPEOOH	80	65	98
PLPSOOMxP	(RB-photo, 24 mg)	10	30	6	4	PLPSOOH	22	14	98
	(UV-photo, 1.0 mg)	10	30	2	4	PLPSOOH	0.9	0.6	93
	(LOX-1, 83 mg)	10	30	12	4	PLPSOOH	78	60	98
ChLOOMxP	(RB-photo, 20 mg)	10	30	6	20	ChLOOH	18	11	98
	(UV-photo, 0.9 mg)	10	30	2	20	ChLOOH	0.8	0.5	95
	(LOX-1, 5.3 mg)	10	30	2	20	ChLOOH	4.8	2.3	95
LLLOOMxP	(RB-photo, 27 mg)	10	30	6	4	LLLOOH	26	19	95
	(UV-photo, 32 mg)	10	30	6	4	LLLOOH	30	21	98
	(LOX-1, 8.2 mg)	10	30	6	4	LLLOOH	7.6	5.0	98
LAOOMxP	(RB-photo, 24 mg)	5	30	1	4	LAOOH	22	14	95
	(UV-photo, 26 mg)	5	30	1	4	LAOOH	24	16	95
	(LOX-1, 85 mg)	5	30	6	4	LAOOH	81	57	98
LAMeOOMxP	(RB-photo, 30 mg)	5	30	1	4	LAMeOOH	28	19	95
	(UV-photo, 33 mg)	5	30	1	4	LAMeOOH	31	20	97
	(LOX-1, 93 mg)	5	30	6	4	LAMeOOH	90	78	>99

^a^Oxidation procedure.

^b^LC retention times and MS profiles of hydroperoxides are as follows; PLPCOOH, 11.0 min, 790.6 [M + H]^+^; PLPEOOH, 10.5 min, 748.5 [M + H]^+^; PLPSOOH, 8.5 min, 790.4 [M − H]^−^; ChLOOH, 26.5 min, 703.6 [M + Na]^+^; LLLOOH, 23.0 min, 933.7 [M + Na]^+^; LAOOH, 15.5 min, 330.3 [M + NH_4_]^+^; LAMeOOH, 11.5 min, 349.3 [M + Na]^+^.

Adapted with permission from Ref. 94. Copyright 2008 American Society for Biochemistry and Molecular Biology.

**Table 8.** Phosphatidylcholine hydroperoxide (PCOOH) (16 : 0/HpODE PC) concentrations in the plasma of healthy subjects and patients with angiographically significant stenosis

MRM	812/541	812/388	812/147^a^
Healthy subjects (pmol/mL, *n* = 8)	36.1 ± 11.5	33.1 ± 10.2	72.3 ± 23.5 (69.3 ± 23.5)
Patients (pmol/mL, *n* = 12)	52.4 ± 24.6	45.2 ± 18.1	97.3 ± 39.5 (97.6 ± 42.2)
*P* value	0.063	0.105	0.127

^a^Contents in parentheses represent the sum of 16 : 0/13-HpODE PC (812/541) and 16 : 0/9-HpODE PC (812/388). Values represent means ± SDs.

Adapted with permission from Ref. 96. Copyright 2015 Elsevier.

**Table 9.** Physical characteristics of Alzheimer’s disease (AD) patients and control subjects

	AD patients	Control subjects
Total number of subjects	10	10
Males	3	4
Females	7	6
Age (years)	80.7 ± 5.8	73.0 ± 5.2
MMSE	21.1 ± 3.5	29.5 ± 0.7
CSF-Aβ (1–42) (pg/mL)	378.4 ± 112.9	560.4 ± 90.5
CSF-tau (pg/mL)	480.5 ± 218.9	209.8 ± 64.2

Means ± SD; *n* = 10.

Adapted with permission from Ref. 111. Copyright 2014 IOS Press.

**Table 10.** Aβ, tocopherol, and phospholipid hydroperoxide in the plasma and red blood cells (RBCs) of patients with Alzheimer’s disease (AD) and control subjects^1^

	Control subjects	Patients with AD
Plasma		
Aβ	(fmol/mL plasma)	
Aβ_40_	81.2 ± 9.8	103.6 ± 11.8
Aβ_42_	18.5 ± 2.8	25.0 ± 5.3
Aβ_42_/Aβ_40_	0.3 ± 0.0	0.3 ± 0.1
Tocopherol	(nmol/mL plasma)	
α-Toc	41.3 ± 3.9	38.4 ± 3.5
Phospholipid hydroperoxide	(pmol/mL plasma)	
PCOOH	29.8 ± 3.7	34.6 ± 5.4
	(µmol/mol phospholipid)	
PCOOH	25.3 ± 4.2	28.9 ± 4.8
RBC		
Tocopherol	(nmol/mL packed cells)	
α-Toc	16.8 ± 2.1	16.3 ± 1.8
Phospholipid hydroperoxide	(pmol/mL packed cells)	
PCOOH	9.6 ± 1.5	44.4 ± 10.4^2^
PEOOH	12.5 ± 2.3	37.6 ± 6.0^2^
PLOOH^4^	22.1 ± 3.6	82.0 ± 13.0^2^
	(µmol/mol phospholipid)	
PCOOH	5.2 ± 0.9	50.7 ± 29.6
PEOOH	6.6 ± 1.2	26.1 ± 7.9^3^
PLOOH	11.8 ± 2.0	76.7 ± 37.3

^1^Means ± SEM; *n* = 18. RBC and plasma PLOOH data were extracted from the PLOOH data (*n* = 28) from our previous study.^116)^

^2,3^Significantly different from control subjects: p < 0.001, p < 0.05.

^4^PLOOH is the sum of PCOOH and PEOOH.

α-Toc, α-Tocopherol; PCOOH, phosphatidylcholine hydroperoxide; PEOOH, phosphatidylethanolamine hydroperoxide; PLOOH, phospholipid hydroperoxide.

Adapted with permission from Ref. 115. Copyright 2016 IOS Press.

## Abbreviations

Aβ: amyloid β-peptide
CL: chemiluminescence
CL-HPLC: chemiluminescence-high performance liquid chromatography
COX-2: cyclooxygenase-2
CSF: cerebrospinal fluid
CSP: chiral stationary phase
DHA: docosahexaenoic acid
DM: type2 diabetes
EGCg: epigallocatechin gallate
Emax: maximum emission
ESR: electron spin resonance
HbA_1c_: glycated hemoglobin
HCC: hepatocellular carcinoma
HCV: hepatitisC virus
HDL: high-density lipoprotein
ICAM-1: intracellular adhesion molecule-1
LC-MS/MS: liquid chromatography–tandem mass spectrometry
LDL: low-density lipoprotein
LFA-1: lymphocyte function-associated antigen-1
LOOH: lipid hydroperoxide
LOX: lipoxygenase
miRNA/miR: microRNA
MRM: multiple reaction monitoring
MS: mass spectrometry
MxP: 2-methoxypropene
nTg: nontransgenic
^1^O_2_: singlet molecular oxygen
PC: phosphatidylcholine
PC-EHP: phosphatidylcholine ethoxyhydroperoxide
PCOOH: phosphatidylcholine hydroperoxide
PE: phosphatidylethanolamine
PEOOH: phosphatidylethanolamine hydroperoxide
PLOOH: phospholipid hydroperoxide
PLPC-OOH: 1-palmitoyl-2-(13-hydroperoxy-*cis*-9, *trans*-11-octadecadienoyl)phosphatidylcholine
PLPE-OOH: 1-palmitoyl-2-(13-hydroperoxy-*cis*-9, *trans*-11-octadecadienoyl)phosphatidylethanolamine
PlsEtn: ethanolamine plasmalogen
POPC: 1-palmitoyl-2-oleoyl-*sn*-glycero-3-phosphocholine
PV: peroxide value
QTRAP: quadrupole/linear ion-trap mass spectrometer
RBC: red blood cell
ROS: reactive oxygen species
SAH: subarachnoid hemorrhage
SQ: squalene
SQOOH: squalene hydroperoxide
TG: triacylglycerol
Tg: transgenic
TGOOH: triacylglycerol hydroperoxide
UV: ultraviolet
